# De Novo Transcriptome Assembly and Gene Expression Profiling of the Copepod *Calanus helgolandicus* Feeding on the PUA-Producing Diatom *Skeletonema marinoi*

**DOI:** 10.3390/md18080392

**Published:** 2020-07-27

**Authors:** Sneha Asai, Remo Sanges, Chiara Lauritano, Penelope K. Lindeque, Francesco Esposito, Adrianna Ianora, Ylenia Carotenuto

**Affiliations:** 1 Integrative Marine Ecology Department, Stazione Zoologica Anton Dohrn, Villa Comunale, 80121 Napoli, Italy; sneha.asai@gmail.com (S.A.); rsanges@sissa.it (R.S.); 2 Area of Neuroscience, Scuola Internazionale Superiore di Studi Avanzati (SISSA), 34136 Trieste, Italy; 3 Marine Biotechnology Department, Stazione Zoologica Anton Dohrn, Villa Comunale, 80121 Napoli, Italy; chiara.lauritano@szn.it (C.L.); esposito@szn.it (F.E.); ianora@szn.it (A.I.); 4 Plymouth Marine Laboratory, Prospect Place, Plymouth PL1 3DH, UK; PKW@pml.ac.uk

**Keywords:** copepods, diatoms, *Calanus helgolandicus*, *Skeletonema marinoi*, transcriptome, oxylipin

## Abstract

Diatoms are the dominant component of the marine phytoplankton. Several diatoms produce secondary metabolites, namely oxylipins, with teratogenic effects on their main predators, crustacean copepods. Our study reports the de novo assembled transcriptome of the calanoid copepod *Calanus helgolandicus* feeding on the oxylipin-producing diatom *Skeletonema marinoi*. Differential expression analysis was also performed between copepod females exposed to the diatom and the control flagellate *Prorocentrum minimum*, which does not produce oxylipins. Our results showed that transcripts involved in carbohydrate, amino acid, folate and methionine metabolism, embryogenesis, and response to stimulus were differentially expressed in the two conditions. Expression of 27 selected genes belonging to these functional categories was also analyzed by RT-qPCR in *C. helgolandicus* females exposed to a mixed solution of the oxylipins heptadienal and octadienal at the concentration of 10 µM, 15 µM, and 20 µM. The results confirmed differential expression analysis, with up-regulation of genes involved in stress response and down-regulation of genes associated with folate and methionine metabolism, embryogenesis, and signaling. Overall, we offer new insights on the mechanism of action of oxylipins on maternally-induced embryo abnormality. Our results may also help identify biomarker genes associated with diatom-related reproductive failure in the natural copepod population at sea.

## 1. Introduction

Diatoms are the dominant component of the marine phytoplankton, being responsible for approximately 50% of primary production in the oceans, and 20% to 25% of all organic carbon fixation on the planet [1]. It has been shown that several diatom species possess a complex infochemical system that plays an important role in allelopathy [2], phytoplankton intercellular communication [3], and phytoplankton bloom termination [4], thus shaping the structure of phytoplankton communities [5]. Several field observations have also demonstrated inhibitory effects of diatoms on the reproduction of calanoid copepods during blooms occurring in the Adriatic Sea [6,7,8], in the English Channel [9], in the Baltic Sea [10], and in the North and South Pacific Ocean [11,12]. Such harmful effects of diatoms on copepod gametogenesis, hatching success, and naupliar fitness, is due to the production of lipid peroxidation compounds termed “oxylipins,” upon cellular wounding, e.g., after copepod grazing. Oxylipins include polyunsaturated aldehydes (PUAs), as well as hydroxyacids, epoxy alcohols, fatty acid hydroperoxides and highly reactive oxygen species (hROS) [13], that may act as defensive metabolites by inducing congenital malformations in copepod offspring and apoptosis in embryos, nauplii and adult females [7,14]. Reduced viability and apoptosis in copepod offspring have been reported either following feeding on oxylipin-producing diatoms (for a review see [15] or after indirect exposure of females to known concentrations of oxylipins [7,16,17]. Recent studies on sea urchin embryos [18] and references therein) and cancer cell lines [19] have highlighted the molecular pathways activated or repressed by PUAs, yet, the unequivocal mode of induction of teratogenesis due to oxylipins in copepods still remains largely unknown.

The calanoid copepod *Calanus helgolandicus* is one of the key copepod species in European waters contributing up to 6% to 93% of the mesozooplankton biomass [20]. The species belongs to the genus *Calanus*, whose members are among the largest copepods and which constitute up to 90% of the mesozooplankton biomass in most marine ecosystems [21]. *Calanus* species play a critical role as grazers of microplankton and prey of juvenile and adult stages of commercially important fish species [22], and also contribute to biogeochemical cycles through vertical migrations [23]. *Calanus helgolandicus* is distributed over a wide range of habitats, from open oceans to coastal environments and is very abundant in the temperate Atlantic Ocean, in the North Sea and in the Mediterranean basin [24,25]. In this area it is considered a “boreal” cold-water species, showing abundance peaks from February to April in both the Adriatic and the Tyrrhenian Seas [20,24]. In recent years, *C. helgolandicus* became a model species for multigeneration cultivation [26] and to investigate copepod molecular responses to oxylipin-producing diatoms, using a gene-target approach and RT-qPCR techniques. In particular, these studies have shown that two days of feeding on the ubiquitous bloom-forming and oxylipin-producing diatom *Skeletonema marinoi*, inhibits a series of genes involved in stress defense, aldehyde detoxification and control of apoptosis in *C. helgolandicus* females [27,28,29]. In another study based on suppression subtractive hybridization techniques and Expressed Sequence Tags (ESTs) libraries, it has been shown that *S. marinoi* activated a generalized cellular stress response (CSR) in *C. helgolandicus* females, by over-expressing genes of molecular chaperones and signal transduction pathways that ultimately protect the copepod from the immediate effects of the diatom diet [30].

These studies provided the first molecular evidence of the harmful effects of oxylipin-producing diatoms on copepods. However, genome-wide approaches such as next-generation transcriptome sequencing could allow the identification of a higher number of transcripts involved in the process, leading to the discovery of the molecular pathways targeted by diatom oxylipins and helping to explain the mechanism of teratogenesis in copepods.

To date, relatively little is known about copepod encoded genes, with genomic resources and RNA-Seq studies limited to a small number of species [31]. Among them, the calanoids *Calanus finmarchicus* [32], *Calanus sinicus* [33]*, Neocalanus flemingeri* [34], *Temora longicornis* [35], and *Acartia tonsa* [36]. In these species, RNA-Seq analysis has been used to generate molecular resources to address physiological and ecological questions such as diapause, lipid biosynthesis, molting and response to abiotic and biotic stressors [32,33,34,35,36,37,38,39] and also to develop molecular markers (SNPs and microsatellites) for studying population genetic diversity [40].

In the present study, we performed a de novo transcriptome assembly and Differential Gene Expression analysis of *C. helgolandicus* females feeding for five days on the oxylipin-producing diatom *S. marinoi*, with respect to a diet of the dinoflagellate *Prorocentrum minimum* that does not produce these compounds. The *S. marinoi* strain used originates from the Northern Adriatic Sea, where it dominates the winter-spring phytoplankton bloom [41] and is reported to produce up to fourteen different oxylipins, including the PUAs heptadienal and octadienal [13]. In order to anchor *C. helgolandicus* molecular responses to direct PUAs ingestion, we also performed gene expression analysis in copepod females exposed for five days to increasing concentrations of heptadienal and octadienal. Specific aims were to: (1) investigate the complete gene expression landscape of *C. helgolandicus* females using transcriptome sequencing technology, (2) characterize the transcriptional profile and identify specific gene targets affected in *C. helgolandicus* females feeding on *S. marinoi*, (3) evaluate quantitative expression of these target genes in females exposed to PUAs. Our ultimate goal was to identify the copepod genes potentially associated with diatom- and oxylipin-induced reproductive failure and teratogenesis, in order to better understand zooplankton physiological ecology, as well as chemically-mediated diatom-copepod interactions at sea.

## 2. Results

### 2.1. Transcriptome Sequencing, De-Novo Assembly and Annotation

RNA samples extracted from *C. helgolandicus* females fed for five days on either *S. marinoi* or *P. minimum* had a mean concentration of 460 ng/μL, RIN ≥ 9, and A260/A230 and A260/A280 ratios ~ 2.0 and were therefore used for cDNA library construction and Illumina sequencing. Illumina-based RNA-Seq of six cDNA libraries in three lanes generated a total of 727 million reads for a total length of 36,324,516,700 bp. After trimming of low-quality reads (quality scores < 20) and short-read sequences (less than 20 bp), a total of 606 million high-quality reads (83.4%) were obtained. The de novo assembly of this 606 million cleaned short reads generated 238,922 transcripts with the average length of 750 bp (N50 = 1114), for a total length of 179,248,227 bp. By splicing and redundancy removing, and filtering out low abundant transcripts, the assembly eventually resulted in 30,339 “Trinity predicted genes” or unigenes (total length = 43,310,775 bp, N50 = 1784 bp and average length = 1427 bp) (Table 1). This represented our *C. helgolandicus* reference transcriptome and contained either singletons as well as the longest isoform of transcripts having multiple isoforms. Minimum and maximum length of sequences was 200 bp and 25,500 bp, respectively, while >50% of unigenes were 1000–5000 bp. The whole size distribution of unigenes is shown in Figure S1.

BLASTx similarity search of 30,339 *C. helgolandicus* unigenes against non-redundant (nr) protein database resulted in 21,336 (70.3% of the total) matched unigenes and 9001 sequences (29.7%) without BLASTx hits, whereas 2 unigenes had only InterProScan exceeding BLASTx size limit of 18,000 bp in Blast2GO. Slightly lower matches were obtained against Swissprot database, with 19,386 matched unigenes (63.9%). The e-value distribution showed that 21.9% and 14.9% of the unigenes have strong homology in the nr database, with BLASTx results ranging from 0 to 1 × 10^−100^ and from 1 × 10^−100^ to 1 × 10^−60^, respectively; whereas 89.4% of the hits have a similarity ranging from 30% to 80% (Figure S2).

The species distribution of the best matches (top-hit) against the nr database of *C. helgolandicus* transcriptome shows that the highest number of matched unigenes have similarities with the copepods *Eurytemora affinis* and *Tigriopus californicus*, followed by other crustaceans, arthropods and copepod species, thus reflecting both phylogenetic relationship and abundant genomic information for those species (Figure S3).

**Figure 1:**
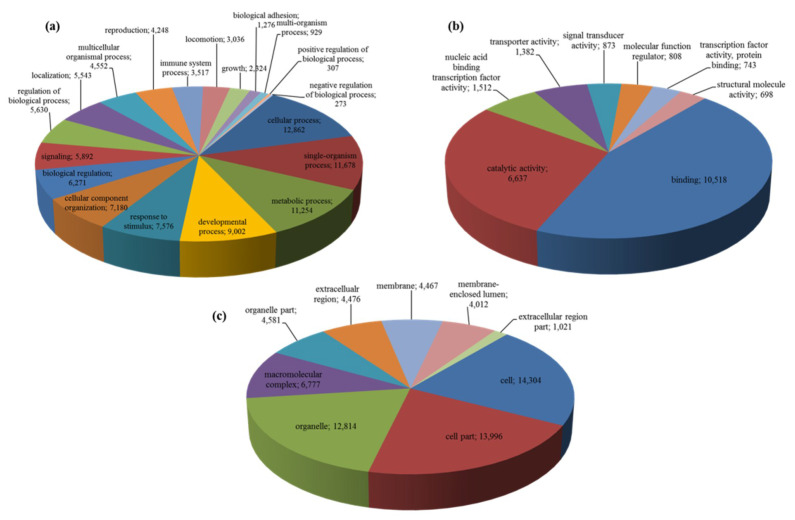
GO categories of annotated unigenes of Calanus helgolandicus transcriptome. GO distribution by level 2 of unigenes assigned to the three main GO categories: (**a**) Biological processes, (**b**) molecular function and (**c**) cellular components. Only top 20 GO terms are shown.

Blast2GO analysis showed that 18,167 unigenes with significant BLASTx matches were functionally annotated (59.9% of the total), receiving in total 376,891 GO terms annotations. These GO terms were distributed as to 231,406 (61.4%) biological processes, 67,273 (17.8%) molecular functions and 78,212 (20.8%) cellular components. It should be noted that multiple GO terms could be assigned to the same unigene. Within the biological process category, cellular process (12,862 out of 18,167 annotated unigenes, 70.8%), metabolic process (11,254 unigenes, 61.9%), developmental process (9002 unigenes, 49.6%), and response to stimulus (7576 unigenes, 41.7%), were among the most represented categories, followed by other important processes such as signaling (5892 unigenes, 32.4%), reproduction (4248 unigenes, 23.4%) and growth (2324 unigenes, 12.8%) (Figure 1a). Within the molecular function category, the highest number of unigenes were assigned to binding (10,518 unigenes, 57.9%) and catalytic activity (6637 unigenes, 36.5%) (Figure 1b). Finally, in the cellular component category, cell (14,304 unigenes, 78.7%) and organelle (12,814 unigenes, 70.5%) were most highly represented (Figure 1c).

There was no difference between the *C. helgolandicus* transcriptome and *Drosophila melanogaster* genome when comparing the proportions of unigenes in the top 20 2nd level GO functional categories, suggesting that the sequenced *C. helgolandicus* transcriptome did not lack any major functional categories of genes (Figure S4).

**Figure 2:**
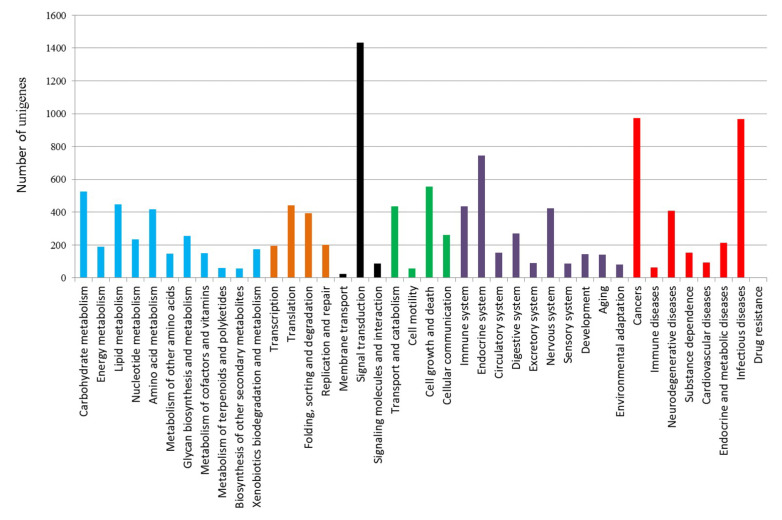
KEGG functional group classification of *Calanus helgolandicus* transcriptome. Number of unigenes assigned to six KEGG functional groups: metabolism (**blue**), genetic information processing (**orange**), environmental information processing (**black**), cellular processes (**green**), organismal systems (**purple**), and human diseases (**red**).

Mapping the 30,339 *C. helgolandicus* unigenes against the KEGG pathway database resulted in 6361 unigenes with assigned KO numbers, for a total of 4710 KO terms associated with the unigenes. Using a pathway reconstruction module, these KEGG annotated unigenes were further categorized into six different functional groups. Specifically, 2663 unigenes were assigned to metabolism (41.9%), 1231 to genetic information processing (19.3%), 1542 to environmental information processing (24.2%), 1312 to cellular processes (20.6%), 2569 to organismal systems (40.4%), and 2872 to human diseases, which was the largest group (45.1%). Interestingly, signal transduction belonging to environmental information processing group was the most represented KEGG category in *C. helgolandicus* transcriptome (22.5%), followed by cancers (15.3%) and infectious diseases (15.2%) (Figure 2). A summary of the statistics of the unigene annotation for *C. helgolandicus* is shown in Table 2.

### 2.2. Differential Expression Analysis

Analysis of expression levels of unigenes showed that 280 sequences were differentially expressed in *C. helgolandicus*, with 117 unigenes significantly up-regulated and 163 unigenes significantly down-regulated in *S. marinoi*-fed compared to *P. minimum*-fed females (FDR ≤ 0.05). All DEGs were further mapped against nr and Swissprot databases, to infer the biological function in which they were involved. Overall, 117 (52 up-regulated and 65 down-regulated) out of 280 DEGs were functionally annotated against nr and Swissprot databases and assigned one or more specific GO term (Table S1). More specifically, in the biological process category, up-regulated DEGs were allocated to a higher number of different but functionally-related GO terms with respect to down-regulated DEGs (74 vs. 47, respectively), of which 88% contained <5 sequences, with respect to 75% for down-regulated genes. This suggested that up-regulated genes encoded for proteins acting in multiple and interconnected metabolic, organismal and cellular processes, such as response to stimulus/stress, protein folding, signaling, biological regulation, lipid metabolic and glutathione metabolic processes (Table S2). In contrast, down-regulated genes showed a lower level of sequence redundancy among terms and were distributed into a more restricted GO list which mainly comprised metabolic and catabolic processes of e.g., organonitrogen compounds (amino acids and nucleotides), proteins, carbohydrates and well-defined amino acids (e.g., serine, glycine and threonine, cysteine, and methionine) (Table S3). It is possible, hence, that the response of *C. helgolandicus* to *S. marinoi* feeding is, on the one hand, the overall activation (up-regulation) of several basic organismal-related and stimulus-related cellular processes, and on the other hand, a reduction (down-regulation) of specific protein—amino acid- and glucose-related metabolic pathways. This output is confirmed by the enrichment analysis on up- and down-regulated gene datasets with respect to all DEGs. The analysis showed that within the biological process category, the GO cellular process (FDR = 6.3 × 10^−3^) and protein folding (FDR = 1.1 × 10^−2^) were significantly enriched in the up-regulated gene set with respect to DEGs. Whereas, GO terms metabolic process (FDR = 1.0 × 10^−4^), organic substance metabolic process (FDR = 2.0 × 10^−4^), primary metabolic process (FDR = 1.3 × 10^−3^), nitrogen compound metabolic process (FDR = 2.2 × 10^−2^) and carbohydrate metabolic process (FDR = 4.3 × 10^−2^), were enriched in the down-regulated unigenes.

**Figure 3:**
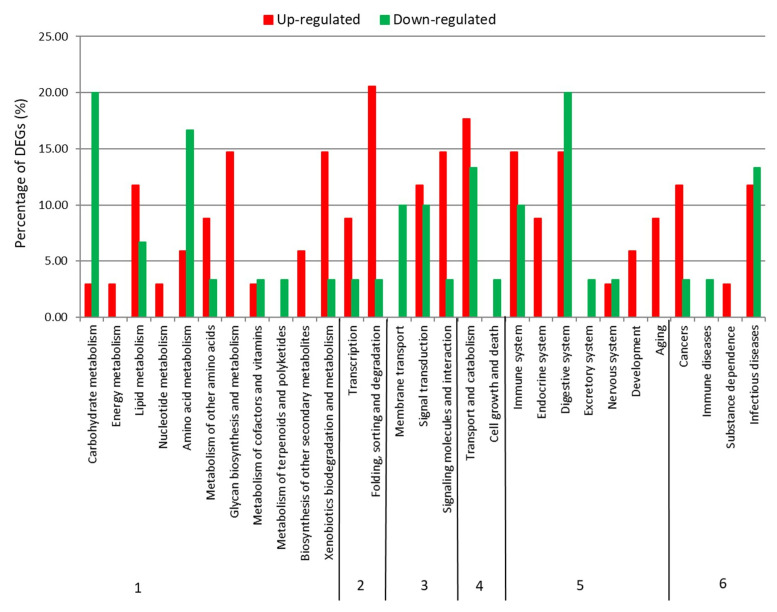
KEGG functional group classification of *Calanus helgolandicus* DEGs. Percentage of up-regulated and down-regulated DEGs assigned to KEGG functional groups within metabolism (**1**), genetic information processing (**2**), environmental information processing (**3**), cellular processes (**4**), organismal systems (**5**), and human diseases (**6**).

KEGG pathway analysis on DEGs resulted in 56 KO groups assigned to 34 up- and 30 down-regulated genes. Distribution of these unigenes within different pathway subcategories reflected previous GO annotation and enrichment results. Up-regulated unigenes, in fact, were mainly allocated to lipid metabolism (12%), xenobiotics biodegradation (i.e., drug metabolism) (15%), folding and degration (i.e., protein processing in endoplasmic reticulum) (21%), signaling (i.e., MAPK signaling pathway and lectins) (15%), transport and catabolism (i.e., endocytosis and lysosome) (18%), immune (i.e., antigen processing and presentation) (15%), endocrine (8%) and digestive (i.e., pancreatic secretion) (15%) systems, aging (9%), and cancers (i.e., chemical carcinogenesis) (12%); whereas down-regulated genes were assigned to carbohydrate (i.e., starch and sucrose) (20%) and amino acid (i.e., glycine, serine, and threonine) (17%) metabolism, membrane transport (i.e., transporters) (10%), and digestive system (i.e., protein digestion and absorption) (20%) (Figure 3).

Overall, the information gathered on GO annotation, KEGG pathway analysis, and enrichment analysis was used for selection of Genes Of Interest (GOIs) belonging to different biological processes to be tested in RT-qPCR analysis. These processes were: response to stimulus/stress, lipid and carbohydrate metabolism, folate and methionine metabolism, embryogenesis, and signaling. Folate and methionine metabolism, named “one carbon pool by folate” in KEGG pathways, was also chosen because, starting from folate, it is involved in the metabolism of nucleotides and amino acids, in particular methionine.

### 2.3. RT-qPCR of Selected GOIs

Thirteen DEGs were chosen to validate RNA-Seq and differential expression results between *C. helgolandicus* females feeding on *S. marinoi* with respect to females feeding *P. minimum*, using RT-qPCR analysis (Table 3). These genes were selected according to FDR value and log2 Fold Change (log2 FC), as well as according to significantly enriched GO terms following the Fisher’s Exact Test. In particular, the following unigenes were chosen: (i) folate and methionine metabolism/one carbon pool by folate: 10-formyltetrahydrofolate dehydrogenase (10-FTHFDH), betaine homocysteine s-methyltransferase 1 (BHMT1) and methylenetetrahydrofolate reductase (MTHFR); (ii) embryogenesis and signaling: vitelline membrane outer layer protein 1 (VMO1), patched domain-containing protein 3 (PTCHD3) and palmitoleoyl-protein carboxylesterase NOTUM (NOTUM); (iii) lipid and carbohydrate metabolism: elongation of very long-chain fatty acids protein AAEL008004 (ELOVL), prosaposin isoform X2 (PSAP), pancreatic triacylglycerol lipase (PTL) and facilitated trehalose transporter 1 (TRET1); (iv) response to stimulus/stress: prophenoloxidase activating enzyme (PPAE), microsomal glutathione s-transferase 3 (MGST3), zinc finger protein OZF-like zinc finger protein OZF-like (OZF).

**Figure 4:**
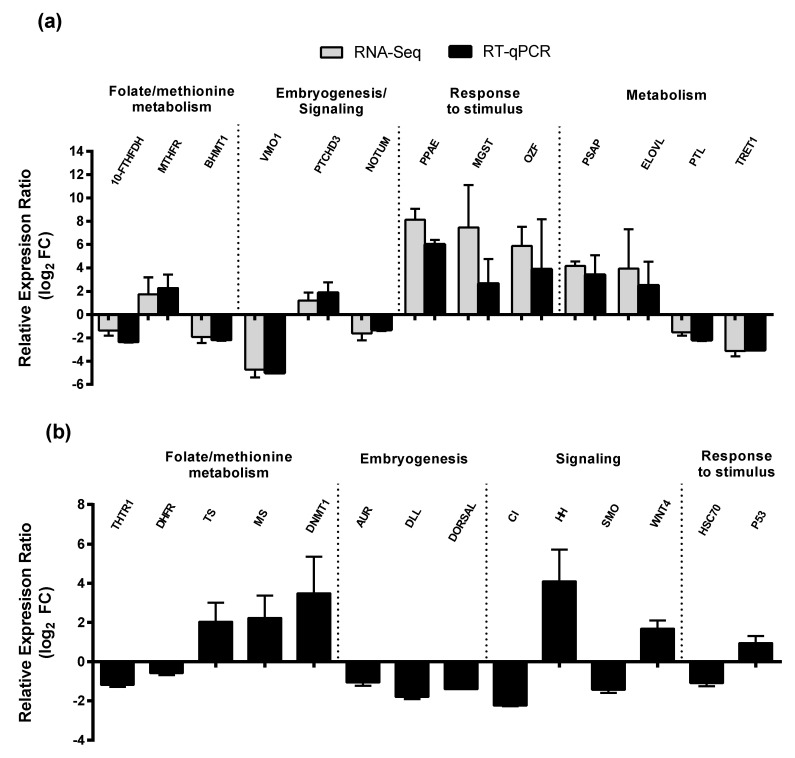
Relative expression ratio (log_2_ FC) of GOIs in *Skeletonema marinoi*-fed *Calanus helgolandicus* versus *Prorocentrum minimum*-fed copepods. (**a**) Comparison of RT-qPCR and RPKM (RNA-Seq) expression values for each GOIs. Gray bars represent RNA-Seq data, whereas black bars represent RT-qPCR data normalized to GAPDH, S20, and EFA. (**b**) RT-qPCR values of additional GOIs belonging to the same biological processes, normalized to GAPDH, S20, and EFA. Bars represent mean ± SD values. All RT-qPCR ratios were statistically significant according to the Pair Wise Fixed Reallocation Randomization Test (*p* < 0.05). Genes were grouped by biological process in which they are involved. For gene abbreviation names see results.

Relative expression ratio of these GOIs in RT-qPCR strongly resembled those obtained from DE analysis (Figure 4a), as indicated by the significant positive correlation between the two datasets (Pearson’s correlation test, r = 0.9421, R2 = 0.888. *p* < 0.0001). More specifically, the three stimulus-related genes were among those that were most strongly up-regulated, with prophenoloxidase activating enzyme (PPAE) being 8-fold and 6-fold up-regulated in RNA-Seq and RT-qPCR analysis, respectively, followed by microsomal glutathione s-transferase 3 (MGST3) (7.5-fold and 2.7-fold, respectively) and the zinc finger protein OZF-like (OZF) (5.9-fold and 3.9-fold, respectively). Other up-regulated genes were involved in metabolism of lipids, PSAP (4-fold and 3-fold, respectively) and ELOVL (4-fold and 2.5-fold, respectively). The most down-regulated gene was the embryogenesis-related protein vitelline membrane outer layer protein 1 (VMO1) (−4.7-fold and −4.5-fold, respectively), as were other sugar- and amino acid metabolism-related genes such as TRET1 (-3-down regulated in both analysis), 10-FTHFDH (−1.4-fold and −2.4-fold, respectively) and BHMT1 (−2-down regulated in both analysis) (Figure 4a).

Relative expression ratio of additional fourteen GOIs within the same GO categories selected from *C. helgolandicus* reference transcriptome, were tested in *S. marinoi*-fed copepods versus *P. minimum*-fed copepods through RT-qPCR (Table 4, Figure 4b).

The results showed that three genes related to folate and methionine metabolism/one carbon pool by folate, such as thymidylate synthase (TS), methionine synthase isoform X1 (MS) and DNA (cytosine-5)-methyltransferase 1 isoform X2 (DNMT1) were all significantly up-regulated in *S. marinoi*-fed versus *P. minimum*-fed females (between 2.0- and 3.5-fold) (Pair Wise Fixed Reallocation Randomization Test, *p* ≤ 0.05), while thiamine transporter 1-like (THTR1) and dihydrofolate reductase isoform X2 (DHFR) were down-regulated (−1.2 and −0.5-fold) (Figure 4b). Similarly, all unigenes related to embryogenesis, aurora kinase B-like (AUR), homeotic protein distal-less (DLL) and embryonic polarity protein dorsal-like isoform X1 (DORSAL), were down-regulated (between −1 and −1.8-fold). For both signaling and response to stimulus, there was no common trend in the selected genes, ranging from −1-fold in the case of heat shock cognate protein 70 (HSC70) and 4-fold for sonic hedgehog protein-like isoform X1 (HH), the remaining genes: transcriptional activator cubitus interruptus-like isoform X2 (CI), protein smoothened (SMO), protein Wnt-4 (WNT4) and cellular tumor antigen p53-like isoform X (P53), included in this range of log2 FC (Figure 4b).

### 2.4. Effect of PUAs on Calanus helgolandicus Gene Expression

**Figure 5:**
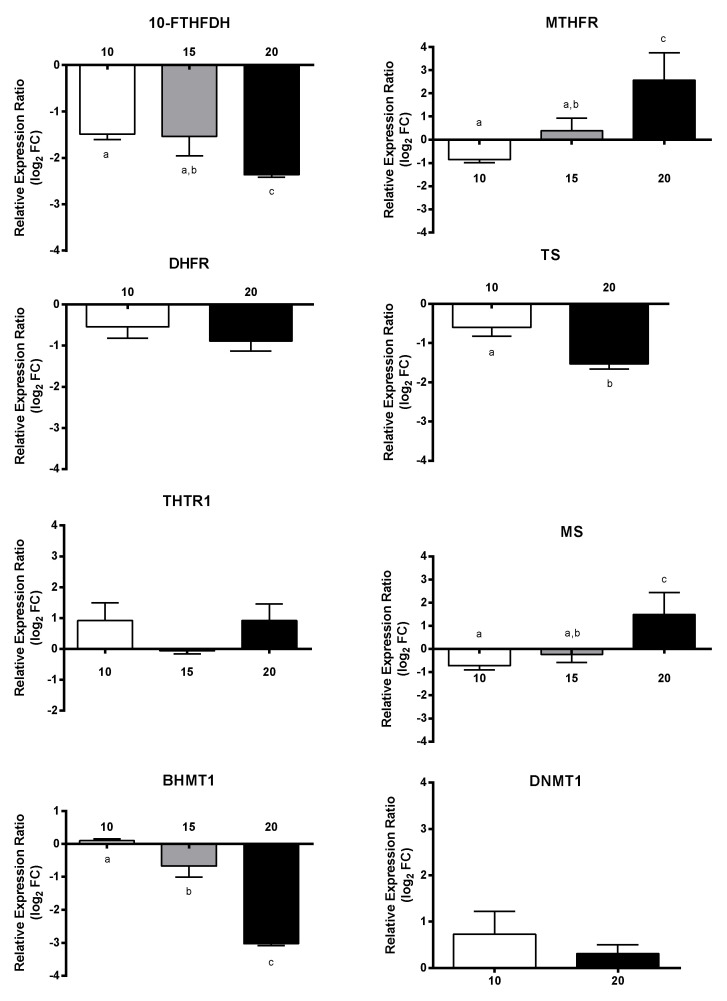
Relative expression ratio (log_2_ fold change) of GOIs related to folate and methionine metabolism in *Calanus helgolandicus* females exposed for five days to a mixed solution of heptadienal and octadienal at a final concentration of 10 µM, 15 µM, and 20 µM, with respect to females exposed to methanol. Values are mean ± SD. Letters a, b and c denoted statistically different treatments after Student’s *t*-test or One-way analysis of variance (ANOVA) for each gene (*p* < 0.05).

**Figure 6:**
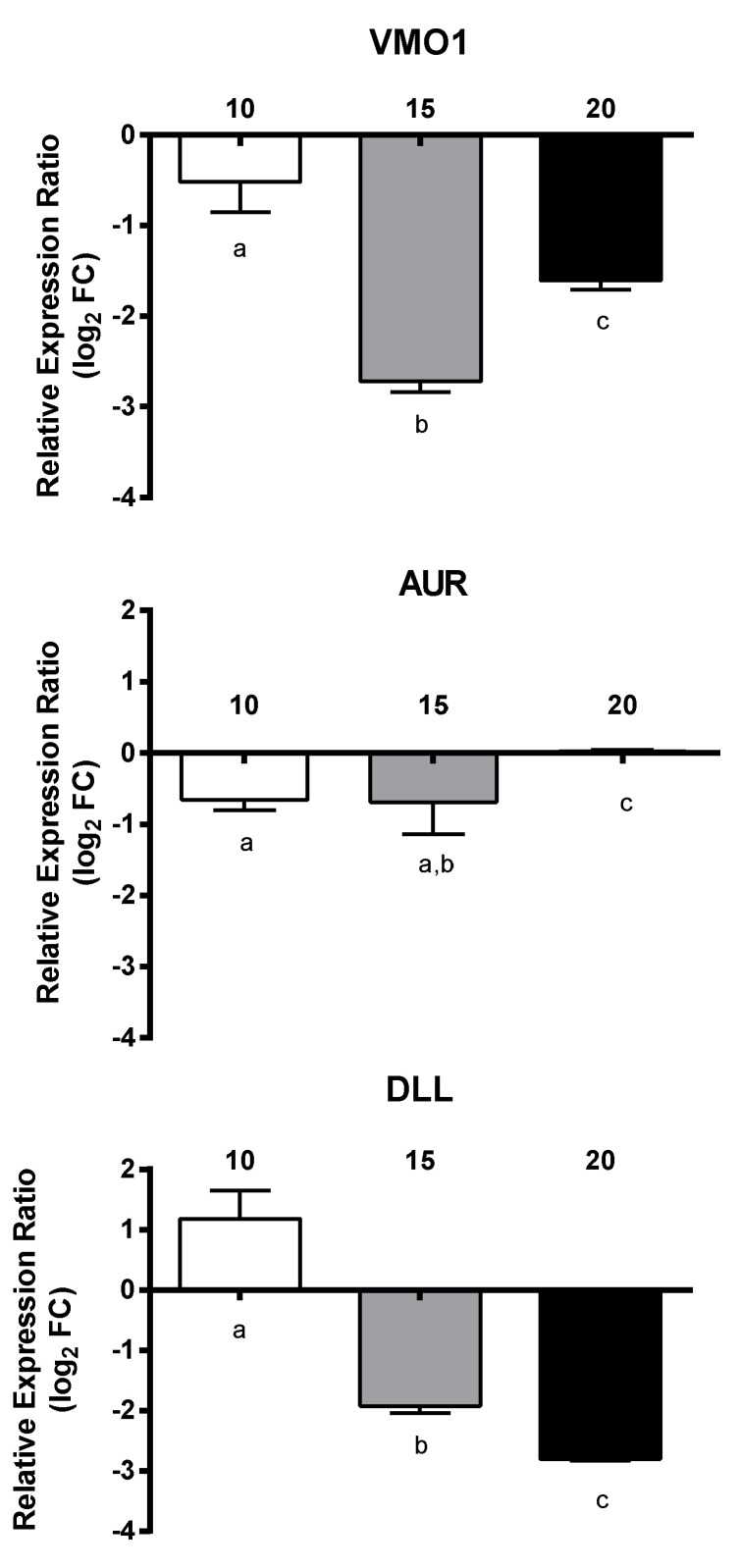
Relative expression ratio (log_2_ fold change) of GOIs related to embryogenesis in *Calanus helgolandicus* females exposed for five days to a mixed solution of heptadienal and octadienal at a final concentration of 10 µM, 15 µM, and 20 µM, with respect to females exposed to methanol. Values are mean ± SD. Letters a, b and c denoted statistically different treatments after Student’s *t*-test or One-way analysis of variance (ANOVA) for each gene (*p* < 0.05).

**Figure 7:**
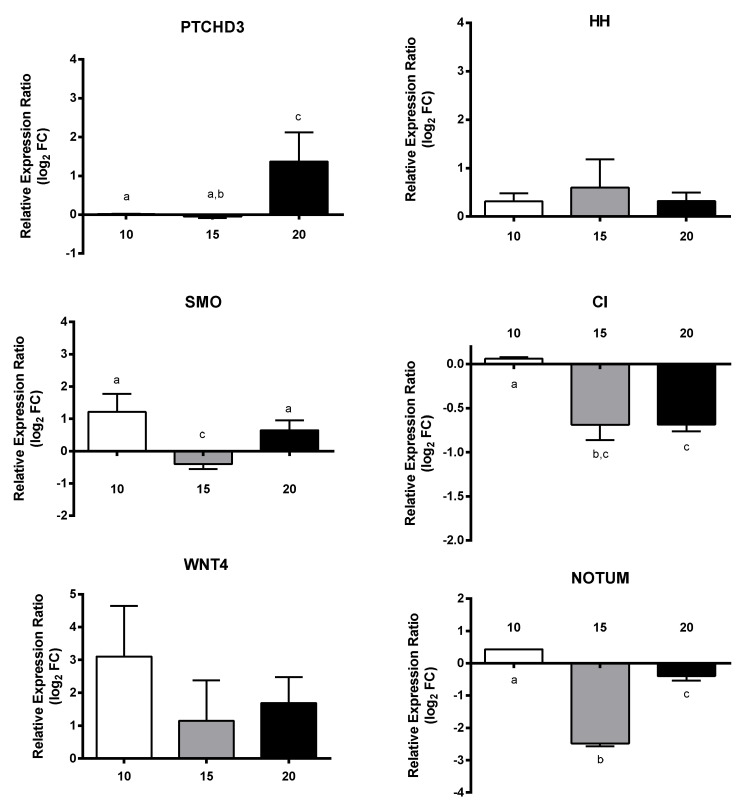
Relative expression ratio (log_2_ fold change) of GOIs related to signaling in *Calanus helgolandicus* females exposed for five days to a mixed solution of heptadienal and octadienal at a final concentration of 10 µM, 15 µM, and 20 µM, with respect to females exposed to methanol. Values are mean ± SD. Letters a, b and c denoted statistically different treatments after Student’s *t*-test or One-way analysis of variance (ANOVA) for each gene (*p* < 0.05).

**Figure 8:**
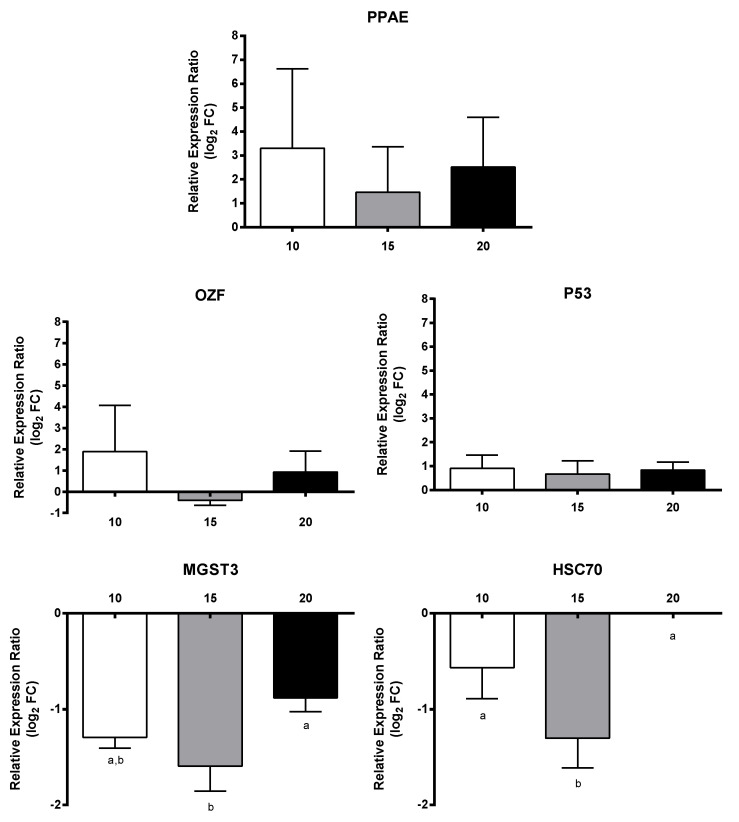
Relative expression ratio (log_2_ fold change) of GOIs related to response to stimulus in *Calanus helgolandicus* females exposed for five days to a mixed solution of heptadienal and octadienal at a final concentration of 10 µM, 15 µM, and 20 µM, with respect to females exposed to methanol. Values are mean ± SD. Letters a and b denoted statistically different treatments after Student’s *t*-test or One-way analysis of variance (ANOVA) for each gene (*p* < 0.05).

**Figure 9:**
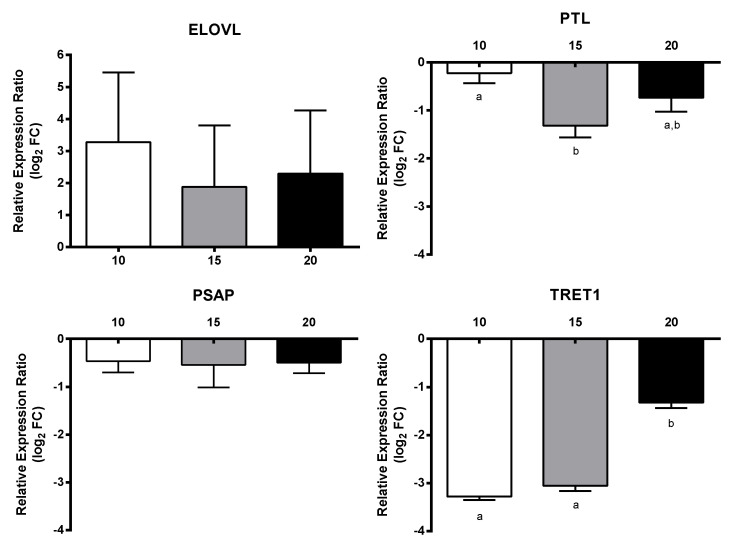
Relative expression ratio (log_2_ fold change) of GOIs related to metabolism in *Calanus helgolandicus* females exposed for five days to a mixed solution of heptadienal and octadienal at a final concentration of 10 µM, 15 µM, and 20 µM, with respect to females exposed to methanol. Values are mean ± SD. Letters a and b denoted statistically different treatments after Student’s *t*-test or One-way analysis of variance (ANOVA) for each gene (*p* < 0.05).

Relative expression ratio of DEGs and other unigenes selected by manual curation, in *C. helgolandicus* females exposed for five days to a mixed solution of heptadienal and octadienal at a final concentration of 10 µM, 15 µM, and 20 µM, with respect to females exposed to control methanol, is shown in Figure 5, Figure 6, Figure 7, Figure 8 and Figure 9. In particular, for unigenes involved in folate and methionine metabolism/one carbon pool by folate, there was a PUAs concentration-dependent decrease in relative expression ratios of 10-FTHFDH, TS and BHMT1, down to a minimum of −3.0-fold, and an increase for MTHFR and MS, up to a maximum of 2.6-fold; whereas THTR1, DHFR, and BHMT1 did not show significant changes (Figure 5).

Regarding transcripts involved in embryogenesis, all three genes VM01, AUR, and DLL were down-regulated, with a dose-dependent decrease only for the latter gene (down to −2.8-fold) (Figure 6). For transcripts involved in signaling, PTCHD3 had a concentration-dependent increase and SMO was up-regulated at 10 µM and 20 µM, whereas CI was down-regulated at 15 µM and 20 µM and NOTUM at 15 µM. The remaining genes WNT4 and HH did not show significant gene expression changes with respect to controls and PUAs concentrations (Figure 7). Among transcripts involved in response to stimulus, only MGST3 and HSC70 were significantly down-regulated (to −1.6-fold) (Figure 8). Finally, genes involved in general metabolism, PTL, and TRET1, were significantly down-regulated, while ELOVL and PSAP did not show significant changes (Figure 9).

## 3. Discussion

The present study reports the first whole-body RNA-Seq analysis of the copepod *Calanus helgolandicus*, generating more than 30 Gb of raw sequence data and 30,339 assembled unigenes. Former studies on *C. helgolandicus* transcriptome analysis were performed using EST sequencing (SSH library) and Sanger sequencing methods, which produced only a few hundred nucleotide sequences [28,30]. As for transcriptome functional annotation, our study indicated that 70.3% (21,337 out of 30,339) of unigenes had homologs in the nr database, much more than those reported for *Calanus finmarchicus* [32], *Temora longicornis* [35], *Acartia tonsa* [36], and *Calanus sinicus* [33]. This could be attributed to the quality of the assembly and/or to the application of more stringent filtering parameters.

GO and KEGG classifications revealed that the assembled *C. helgolandicus* unigenes have diverse molecular functions and are involved in many metabolic and cellular pathways, thus reflecting gene richness of the global landscape of the transcriptome. Overall, the highest number of annotated unigenes were involved in cellular and metabolic processes, developmental processes, response to stimulus, and signaling, similar to that which has been reported previously [30,32,35,37]. Interestingly, the KEGG pathway classification analysis showed the highest number of unigenes was classified into signal transduction and human diseases, especially infectious diseases. Aquatic organisms may often have to deal with the challenges imposed by parasitic, bacterial, and viral infections in water; thus the presence of such signal transduction pathways in copepod transcriptomes could have evolved as a defense mechanism against infection, as suggested for the white-leg shrimp *Litopenaeus vannamei* [42].

Our differential expression analysis of *C. helgolandicus* females following an oxylipin-producing diatom diet was helpful for the identification of candidate genes underlying the response of *C. helgolandicus* to the diatom diet and their PUAs. In particular, within the 280 differentially expressed unigenes between *Skeletonema marinoi*-fed and *Prorocentrum minimum*-fed *C. helgolandicus* females, several key processes and/or metabolic pathways, such as response to stimulus/stress (e.g., detoxification mechanism), protein folding, signal transduction, transport, immune response, carcinogenesis, carbohydrate, lipid, protein, and aminoacid/tetrahydrofolate metabolism, were significantly altered in copepod females after five days of feeding on *S. marinoi*. More importantly, incubation experiments with pure PUAs supported results obtained during copepod feeding on the diatom, suggesting that the observed molecular responses were related to the ingestion of oxylipins and their direct impairment or stimulation of specific gene expression. This is the first study reporting the molecular effects of the diatom PUAs octadienal and heptadienal on copepods and will help to link molecular responses to phenotypic responses observed in copepods due to oxylipin-producing diatoms. Although the concentrations of PUAs in the incubation experiment are higher than the concentrations theoretically offered to the copepods in the feeding experiment, considering the daily algal supply of 45.000 cells mL^−1^ and the potential PUAs production of 2 fmol cell^−1^ measured in the same *S. marinoi* strain used in our study [43], for a total of about 0.1 µM of PUAs, it has to be pointed out that quantification of oxylipins in algal cultures is always a potential production, based on a fixed incubation time during which free fatty acids are converted into oxylipins. On the contrary, oxylipin production in the copepod is an active process occurring continuously in the copepod gut, starting from algal ingestion, crushing of cells, and release of the oxylipins. It is, therefore, possible that the actual amount of oxylipins ingested by the copepod during the feeding experiment is higher than the potential production by the algal culture. Moreover, concentrations of 10–20 µM of PUAs induced the same reduction of egg viability and increase of naupliar abnormality in *C. helgolandicus* embryos as observed during algal feeding experiments with *S. marinoi* [13].

The activation of drug (xenobiotics) metabolism and detoxification systems is one of the most common responses to exogenous chemical compounds. In this study, several sequences similar to detoxification system genes were up-regulated in *C. helgolandicus* following feeding of *S. marinoi*, including microsomal glutathione s-transferase 3 (MGST3), pyrimidodiazepine synthase and xanthine dehydrogenase/oxidase. The same was observed for several genes coding for proteins involved in generic stress/stimulus response and protein folding, such as prophenoloxidase activating enzyme (PPAE), zinc finger protein OZF-like (OZF), heat shock cognate protein 70 (HSC70), E3 ubiquitin-protein ligase SINAT3 and dnaJ protein homolog 1. These results were confirmed by the KEGG pathway reconstruction of up-regulated genes which showed high percentages in folding, sorting and degradation, xenobiotics biodegradation and chemical carcinogenesis, the latter likely associated with the shared reactive functional group of heptadienal and octadienal with malondialdehyde and 4-hydroxy-2-nonenal, well-known PUAs having carcinogenic activity [44].

Interestingly, our findings differed from those described in [28], which reported down-regulation of genes involved in stress response and defense (glutathione S-transferase and cytochrome P450 enzymes) in *C. helgolandicus* feeding on the same *S. marinoi* strain. This could be related to the shorter feeding period as compared to the present experiment (2 days vs. 5 day), thus suggesting that prolonged ingestion of the diatom might induce stronger toxic effects eliciting activation of detoxification systems. Up-regulation of genes associated with repair/degradation systems in the present study confirm findings by [30] that *S. marinoi* induced over-expression of genes involved in protein folding or degradation in *C. helgolandicus*, thus protecting the adult copepod from the direct toxic effect of the diatom diet. Cellular chaperones (i.e., heat shock proteins) can transfer irreparably damaged proteins for degradation through the ubiquitin–proteasome pathway [45]. Similar up-regulation of transcripts encoding proteins involved in the ubiquitin–proteasome pathway has also been shown in the copepod *C. finmarchicus* following exposure to diethanolamine [46]. Our incubation experiments with pure PUAs in general confirmed results induced by the diatom diet, with 2–3-fold up-regulation of PPAE and OZF, although opposite trends were observed for MGST3 and HSC70. It is possible that the amount of PUAs indirectly ingested by copepod females in this in vitro test was too low to induce the activation of such a detoxification system, thus resembling shorter feeding trials [28].

The gene showing the highest up-regulation in *C. helgolandicus* females feeding on *S. marinoi* was the prophenoloxidase activating enzyme PPAE (8-fold), an enzyme with endo-peptidase activity involved in proteolysis. In crustaceans, activation of prophenoloxidase, together with pattern recognition proteins, complement, and coagulation cascade, is part of the organism immune response [47]. In the current study, the sequence complement C1q tumor necrosis factor-related protein 3-like was part of the up-regulated gene set, which also showed a high number of sequences allocated to KEGG pathway category “antigen processing and presentation,” related to the immune system. We also identified in the whole transcriptome transcripts belonging to pattern recognition protein lectins (c-type lectin domain family 4 member k). A similar immune response was reported for the Chinese shrimp *Fennropenaeus chinensis* during white spot syndrome virus (WSSV) acute infection [48] and in white shrimp *Litopenaeus vannamei* exposed to nitrite [49]. Hence, these immune response-related genes can be activated as part of the defensive system of the copepod to cope with the harmful effects of a diatom diet, potentially associated with lipid peroxidation production induced by PUAs and other oxylipins.

Overall, our results suggest the occurrence of a mechanism of transient impairment of early stress defense response in *C. helgolandicus* females, induced by short-term feeding on oxylipin-producing diatoms (2 days), followed by later activation of detoxification, protein repair, and immune systems genes during longer feeding on the diatom diet (5 days). This concerted response might explain the high *C. helgolandicus* female survival observed in the present study (data not shown) and also reported in previous laboratory feeding experiments over fifteen days [7].

Ingestion of *S. marinoi* and exposure to PUAs also led to deregulation of folate and methionine metabolic processes, as well as of signaling pathways involved in oogenesis/embryogenesis, cellular proliferation and patterning, possibly leading to reduced embryo viability, teratogenesis, and apoptosis.

Folate (vitamin B9), is an essential cofactor for the de novo synthesis of purines and pyrimidines and hence DNA synthesis, methylation of DNA and proteins, through one-carbon metabolism and methionine cycle, respectively [50]. This complex metabolic pathway is well known in vertebrates, where its alteration is associated with increased risk for neural tube defects and other transgenerational developmental defects [51,52]. In arthropods, altered folate metabolism is associated with abnormal body patterning in *Drosophila* [53] and *Artemia* larvae [54]. Additionally, maternal exposure to the folate inhibitor drug methotrexate produced leg and wing deformities in surviving progeny of *Drosophila* [53,55]. To date, the genes involved in folate metabolism are not characterized in crustaceans, except for thymidylate synthase (TS) in *L. vannamei* [56]. Therefore, this is the first report of folate metabolism genes in a planktonic crustacean.

Our data showed that *C. helgolandicus* females feeding on *S. marinoi* or incubated with pure PUAs, had decreased expression level of genes encoding for 10-formyltetrahydrofolate dehydrogenase (10-FTHFDH) and dihydrofolate reductase (DHFR), both involved in replenishment of tetrahydrofolate (THF), the active form of folate [56]. Reduced expression of these genes might lead to decreased levels of THF and, hence, of downstream substrates for the production of 5,10-methylene THF, the donor cofactor for the transfer of one carbon units for purine and pyrimidine biosynthesis catalyzed by TS [57]. Inhibition of DHFR activity in zebrafish has been linked to occurrence of abnormal embryos [51], whereas 10-FTHFDH knock-out in the embryos, lead to delayed early development and subsequent anomalies [58]. *Calanus helgolandicus* females feeding on *S. marinoi* and exposed to PUAs also showed up-regulation of the methylenetetrahydrofolate reductase (MTHFR) gene, which is responsible for the irreversible conversion of 5,10-methylene THF into 5-methyl THF. This substrate is converted back into THF by the methionine synthase (MS), via methylation of homocysteine into methionine, thus linking the one carbon folate cycle to the methionine cycle and, hence, to DNA methylation [56]. Over expression of MTHFR in *C. helgolandicus*, therefore, might increase shuttling of 5,10-methylene THF towards the methionine cycle at the expense of purine and pyrimidine biosynthesis. Reduced conversion of dUMP to dTMP has been associated with increased uracil misincorporation, DNA damage, and apoptosis in humans [59]. In addition, treatment of mouse embryos with the known teratogen valproic acid induced an increase in MTHFR gene expression and was associated with higher risk of congenital malformations [60]. In concert with higher expression of MTHFR gene we also observed up-regulation of MS gene, probably as a response to higher substrate availability, and down-regulation of betaine-homocysteine methyltransferase 1 (BHMT), a likely compensatory mechanism to re-equilibrate methionine levels, being the enzyme responsible for the methylation of homocysteine into methionine using the amino acid betaine [57]. The potential effect of *S. marinoi* and PUAs on the methylation cycle is further supported by the up-regulation of DNA methyltransferase 1 (DNMT1) gene, involved in the methylation of cytosine residues, possibly leading to increased methylated products. DNA hyper-methylation may affect epigenetic control of gene transcription and, in turn, embryogenesis and development [61].

Taken together, our results suggest that the adverse effects on *C. helgolandicus* oocytes and developing embryos, induced by maternal exposure to diatoms and oxylipins PUAs [7], could be due to an altered folate and methionine metabolism in the females. Folate-dependent reactions are, in fact, essential for early growth and development in arthropods [62]. Since PUAs accumulate selectively in copepod gonads [63], it is possible that diatom oxylipins may directly target reproductive tissues and, in turn, affect embryogenesis [64].

The present study also showed that several transcripts involved in oogenesis, vitellogenesis, and developmental signal transduction pathways, were modulated in *C. helgolandicus* females after diatom feeding and direct PUAs incubation. For example, strong down-regulation was observed in both *S. marinoi*-fed and PUAs-exposed females of the gene coding for the vitelline membrane outer layer protein 1 homolog (VMO1). The vitelline membrane plays a major role in preventing mixing of yolk and albumen in eggs, whose incomplete separation has been associated with the production of abnormal oocytes and delayed development [65]. It also provides positional information to the developing embryos, being the spatial repository for dorso-ventral and antero-posterior embryonic patterning determinants produced by follicle cells during oogenesis [66,67]. In crustaceans, VMO1 proteins are synthesized by extraovarian tissues and then transported via the hemolymph to the developing oocytes [65]. Its role is still not fully known, although it has been suggested that altered expression of this gene in *Daphnia magna* females exposed to ibuprofen, was associated with failed oogenesis, abnormal oocytes possibly being re-absorbed and blocked embryogenesis [68]. It is therefore possible that reduced expression of VMO1 genes could play a role in the arrested oocyte maturation and oocyte degradation previously observed in *C. helgolandicus* females feeding on oxylipin-producing diatoms [69].

In most animals, the early embryonic development depends on maternally provided mRNA, which is crucial for cell cycle progression, axis patterning, and cell fate specification events [70]. Hence, maternally encoded transcripts might play a role in diatom-derived effects on *C. helgolandcus*. The aurora A kinase (AUR) is a maternally supplied cell cycle regulator which plays an important role in cell-cycle progression of fertilized eggs and early embryos of mouse [71]. Silencing of AUR gene leads to asynchronous mitotic cycles and fusion of mitotic spindles leading to larval lethality [72]. Similarly, the maternal transcript distal-less (DLL) functions as a homeodomain transcription factor and plays one of the major roles in limb development throughout the animal kingdom [73]. DLL has been found in insects and crustaceans, where it specifies distal structures and promotes outgrowth of the segmented appendages [74]. Finally, the *Drosophila* gene dorsal (DORSAL) is a maternal transcription factor essential for the establishment of dorsal-ventral polarity in the developing embryo [75]. Overall, down-regulation of these maternally-encoded transcription factors in *C. helgolandicus* females feeding on *S. marinoi* and exposed to PUAs, might thus contribute to impaired embryonic development of early non-feeding nauplii.

As for the signal transduction Hedgehog pathway, this developmental pathway plays a key role in invertebrate development and early embryogenesis [76]. In *Drosophila*, hedgehog signaling controls patterning of imaginal disc-derived adult structures such as appendages [77]. In the present study, we observed up-regulation of the gene encoding for the extracellular ligand sonic hedgehog protein-like isoform X1 (HH), homologous to hedgehog in *Drosophila*, and this matched the increase in expression level of the gene encoding for its receptor, patched domain-containing protein 3 (PTCHD3). However, the down regulation of the downstream cytoplasmic signal transducer smoothened (SMO) and transcription factor cubitus interruptus (CI) genes, suggests possible down-regulation of Hedgehog signaling pathway in *C. helgolandicus* after five days feeding on *S. marinoi* and PUAs incubation. This is also supported by the observed down-regulation of the DLL gene, one of the targets of Hedgehog. Reduction of DLL activity causes defects of distal leg segments in arthropods, including insects [78] and the crustaceans *Parhyale hawaiensis* [79], *Daphnia magna* [80] and *Daphnia pulex* [81].

**Figure 10:**
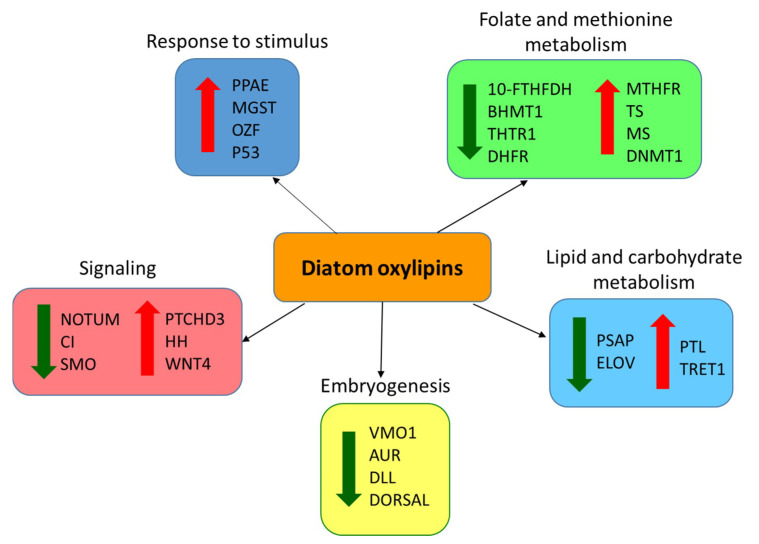
Summary of genes and biological processes affected in *Calanus helgolandicus* after ingestion of *Skeletonema marinoi* and/or exposure to oxylipins PUAs. For gene abbreviation names see results.

In conclusion, the present study indicates that maternal ingestion of *S. marinoi* and exposure to PUAs by *C. helgolandicus* for five days leads to de-regulation of folate metabolism, possibly leading to decreased DNA synthesis and altered gene methylation, as well as disturbance of oogenesis/embryogenesis signaling pathways involved in cellular proliferation and patterning, thus, causing reduced embryo viability and teratogenesis. A summary of the genes and processes affected in *C. helgolandicus* is depicted in Figure 10. These results add new insights to the chemically-mediated ecological interactions between diatoms and copepods, providing novel information on the molecular mechanism of action of oxylipins on maternally-mediated teratogenesis of copepod embryos. Our results will also prompt further exploration of the molecular effects of less studied oxylipins, such as hydroxyacids, epoxyalcohols, and fatty-acid hydroperoxides, on copepod reproduction. These oxylipins have been found to impair copepod reproduction in the laboratory by inducing naupliar apoptosis similar to PUAs [7,13]. Recently, reduced hatching success and increased expression of stress-related genes have also been measured in *C. helgolandicus* females collected during diatom blooms in the Northern Adriatic Sea, when high oxylipin concentrations were measured in the natural phytoplankton assemblage [8]. Therefore, our results will also contribute to the evaluation of the expression of new biomarker genes associated with diatom-related reproductive failure in the natural copepod population at sea.

## 4. Materials and Methods

### 4.1. Phytoplankton Culture

The centric diatom *Skeletonema marinoi* (strain FE6) and the dinoflagellate *Prorocentrum minimum* (strain FE100), were grown in f/2 medium and K medium, respectively. Phytoplankton cultures were grown as semi-continuous batch cultures to late-exponential phase of growth in 2-L glass jars kept in a temperature-controlled room under 18 °C, on a 12:12 Light: Dark cycle and an irradiance of 100 μE m^−2^ s^−1^.

### 4.2. Copepod Collection and Feeding Experiments

*Calanus helgolandicus* specimens were sorted from zooplankton samples collected in the Gulf of Naples during May 2012 by using 200 μm Nansen net in oblique tows. Adult *C. helgolandicus* females were isolated under a Leica stereomicroscope, transferred to 1L jars (*n* = 20–30 copepods) filled with 0.22 µm filtered sea water (FSW) and enriched with either *S. marinoi* (45,000 cells mL^−1^, 1 mg C L^−1^), or *P. minimum* (5000 cells mL^−1^, 1 mg C L^−1^). Jars were kept in a temperature-controlled room under 18 °C, on a 12:12 Light: Dark cycle. The algal medium was changed daily with addition of fresh FSW and either *S. marinoi* or *P. minimum* diet. After five days, the copepods were transferred to clean jars containing 0.22 µm FSW for 24 h in order to allow gut evacuation. Three independent experiments were performed on different occasions during May 2012, each one with both the diatom and the control treatment and were considered as biological replicates. Ten females per replicate diet were transferred into 1.5 mL Eppendorf tube containing 500 µL of RNAlater^®^ reagent and processed according to manufacturer’s instructions.

### 4.3. Transcriptome Sequencing

Total RNA was extracted from *C. helgolandicus* females using the RNeasy Micro Kit (Qiagen, Germany) following manufacturer’s procedure [82]. RNA concentration (ng µL^−1^) and purity were assessed through Nanodrop ND-1000 UV-Vis spectrophotometer (Marshall Scientific, Hampton, NH, USA), whereas RNA Integrity Number (RIN) was checked on a 6000 Nano LabChip of Agilent Bioanalyzer 2100 (Agilent Technologies, Santa Clara, CA, USA). A total of 3 µg of RNA per sample (300 ng µL^−1^) was delivered to the Genomics Core Facility of the European Molecular Biology Laboratory (EMBL, Heidelberg, Germany), for library preparation using TruSeq RNA Sample Prep Kit (Illumina, San Diego, CA, USA). Fragments >200 bp were selected, purified and subsequently PCR amplified to create the final cDNA library template for sequencing. RNA-Seq was conducted on Illumina HiSeq 2000 platform with 50 bp paired end option. Two cDNA libraries obtained from copepods fed with either *S. marinoi* or *P. minimum* from one replicate experiment, were sequenced individually in two single lanes of the Illumina flow cell to obtain a deep and high qualitative coverage of *C. helgolandicus* transcriptome. The other four cDNA libraries obtained from copepods fed with either *S. marinoi* or *P. minimum* during two other independent experimental replicates were used for multiplexed sequencing in one single lane of an Illumina flow cell.

The Raw Reads generated are publicly available in the NCBI Sequence Read Archive (SRA) repository (accession number PRJNA64,0515; https://www.ncbi.nlm.nih.gov/sra/PRJNA640515).

### 4.4. De novo Transcriptome Assembly and Functional Annotation

Cleaning, trimming, quality filtering and removal of the adapters was performed with the program Trimmomatic [83] combined with custom scripts [84] with the following options: ILLUMINACLIP set at 2:40:15 with the use of standard Illumina adapters sequences used as filter, LEADING and TRAILING set at 5, SLIDINGWINDOW at 5:20 and MINLEN at 30 (Script can be found here: https://github.com/silverkey/transcriptome/blob/master/preprocess_fastq_for_trinity.pl). The remaining high quality paired-end reads across the six samples were assembled using Trinity [85] (version 2013-02-25) with default plus the following parameters: --seqType fa --JM 200G --inchworm_cpu 20 --bflyHeapSpaceInit 20G --bflyHeapSpaceMax 200G --bflyCalculateCPU --CPU 20 &. This ‘reference’ *C. helgolandicus* transcriptome consisted of de novo assembled Trinity transcripts with unique TR#_c#_g# identifiers (‘Trinity predicted genes’ or unigenes) and contained either singletons (transcripts with a single isoform, ‘i’) as well as the longest isoform of transcripts having multiple ‘Trinity predicted isoforms’ (TR#_c#_g#_i#) [86]. To exclude contaminations, poorly supported transcripts and artifacts, the assembled unigenes were further filtered based on a minimum expression level of more than 1 read per million mapped reads (RPKM) in at least 2 samples.

Unigenes were further assigned a putative protein function by a sequence similarity search using the BLASTx algorithm (e-value ≤ 10^−3^) against the NCBI non-redundant protein sequence database (nr) and Swissprot database, followed by Gene Ontology (GO) functional annotation (e-value ≤ 10^−6^), using Blast2GO PRO version 5 [87]. The Web Gene Ontology Annotation Plot (WEGO) software [88] was then used to plot and compare the GO annotation results among related organisms. Additionally, Kyoto Encyclopedia of Genes and Genomes (KEGG) Automatic Annotation Server (KAAS) database [89] was also searched using BLASTx algorithm (e-value ≤ 10^−5^), to provide information about metabolic pathways in the dataset.

### 4.5. Differential Gene Expression Analysis

To identify differentially expressed unigenes between *S. marinoi*-fed and *P. minimum*-fed *C. helgolandicus* cDNA libraries, normalized raw reads from each replicate feeding treatment were firstly mapped back on the reference transcriptome using BOWTIE [90] and then counted using RSEM software [91] to estimate the gene expression level (https://github.com/silverkey/transcriptome/blob/master/launch_mapping_trinity_analysis_folder.pl). The three replicate samples from each treatment were used to generate mean expression levels using RPKM method. Statistical analysis was performed using R/Bioconductor and the EdgeR package to identify differentially expressed genes (DEGs). Significance values were obtained by performing a hypergeometric test and corrected *p*-value using the false discovery rate (FDR) method [92]. Genes having a FDR ≤ 0.05 were considered differentially expressed and further annotated against nr and Swissprot using Blast2GO, and against KEEG database using KAAS, according to the procedure described for the reference transcriptome. Functional enrichment analysis for up- and down-regulated genes in *S. marinoi*-fed versus *P. minimum*-fed *C. helgolandicus* females, with respect to all DEGs, was also performed by Blast2GO using the Fisher’s exact test with multiple testing correction of false discovery rate (FDR ≤ 0.05).

### 4.6. RT-qPCR of Genes of Interest (GOIs)

A series of functionally annotated genes of interest (GOIs) were selected from the list of DEGs according to their FDR and log2 fold change and used for validation of RNA-Seq analysis through real time-quantitative PCR (RT-qPCR). Other functionally annotated GOIs were also selected from the de novo assembled transcriptome, according to their putative involvement in the response of copepods to diatoms and tested by RT-qPCR. Specific forward and reverse oligonucleotide primers were designed using Primer3 software (v. 0.4.0) as in [27] and synthesized by Primm Labs (Milan, Italy).

A panel of seven putative reference genes (RGs), previously optimized in *C. helgolandicus* [27], were screened to identify the most stable genes in the present experimental conditions: elongation factor 1a (EFA), histone 3 (HIST3), glyceraldehyde-3-phosphate dehydrogenase (GAPDH), ribosomal units (18S, S7, S20) and ubiquitin (UBI). The web-based comprehensive tool RefFinder (http://www.leonxie.com/referencegene.php) was used for evaluating the most stable reference genes using Ct values as direct input. Based on these rankings, the best RGs were EFA, GAPDH and S20.

RT-qPCR reactions of reference genes as well as GOIs, were performed in MicroAmp Optical 384-Well reaction plate (Applied Biosystem, Foster City, CA, USA) with optical adhesive covers (Applied Biosystem), using a Viia7 real-time PCR system (Applied Biosystem, Foster City, CA, USA). First, cDNA template was retrotranscribed from 1 μg of total RNA remaining from RNA-Seq analysis, using iScriptTM cDNA Synthesis Kit (BIORAD) and following the manufacturer’s instructions, in the GeneAmp PCR System 9700 (Applied Biosystems). cDNA template (1 μL at 1:100 dilution) was then mixed with 5 μL of Fast Start SYBR Green Master Mix (Applied Biosystem) and 0.7 pmol/μL of each primer, for a final PCR sample volume of 10 μL. RT-qPCR reactions were carried out in triplicate, including at least two no-template negative controls for each primer pair, using PCR conditions previously reported in [30]. Serial dilutions of cDNA (1, 1:5, 1:10, 1:50, 1:100 and 1.500) were used to calculate reaction efficiencies (E) for all primer pairs using the equation E = 10^−1^/slope from a standard curve between Ct values and the log10 for each dilution factor. The relative expression ratio (R) of each GOI in the experimental conditions (copepods fed on *S. marinoi*) versus the control condition (copepods fed on *P. minimum*) and its statistical significance was calculated using REST (Relative Expression Software Tool) and the Pair Wise Fixed Reallocation Randomization Test [93]. The calculation is based on the efficiencies (E) and the Ct deviation between the experimental and the control groups of the target genes normalized to the reference genes EFA, GAPDH and S20, and is expressed as log2 fold change. Differences were considered significant when Randomization Test *p*-value was ≤0.05.

### 4.7. PUAs Incubation Experiments and RT-qPCR of GOIs

*Calanus helgolandicus* adult females were collected in the Gulf of Naples during March–May 2013 and fed with *P. minimum* (5000 cells mL^−1^) before the start of the experiment. Stock solutions of the polyunsaturated aldehydes (PUAs) 2-trans,4-trans-heptadienal and 2-trans,4-trans-octadienal (Sigma-Aldrich, Italy) at 10 mM and 1 mM were prepared by diluting the proper amount of PUAs in absolute methanol (J.T. Baker, Holland). Final working solutions were obtained by diluting stock solutions in FSW and mixing carefully to ensure optimal distribution. Methanol has no negative effect on copepod females and eggs up to 1% methanol in seawater [7]. The amount of aldehyde solution in each test concentration was kept below this threshold (final concentration used: 0.2% of methanol). PUA incubation experiments were performed by incubating individual *C. helgolandicus* female (*n* = 5−7) in 100-mL crystallizing dishes containing *P. minimum* (5000 cells mL^−1^) and 1:1 PUA mixture of heptadienal + octadienal (MIX) at final concentration of 10–15–20 μM in FSW. Females incubated in *P. minimum* + methanol were used as controls. Females were kept at 18 °C in a controlled temperature chamber under 12:12 Light: Dark cycle and transferred to new crystalizing dishes with fresh PUA mixture and algal diet daily. After 5 days of incubation, females were placed in 500 μL RNAlater^®^ (5–7 females per treatment), frozen according to the manufacturer’s instructions and stored at −80 °C until RNA extraction. A total of 3 experiments were performed during March–May 2013. Extraction of total RNA, retrotranscription of cDNA and RT-qPCR analysis was performed as previously described. Stability of reference genes was screened again in copepods exposed to PUAs by RefFinder and the most stable genes identified were S20, GAPDH and UBI. Relative expression ratio (log2 fold change) of the same GOIs selected previously were calculated between *C. helgolandicus* females incubated in each PUAs MIX concentration (experimental condition) and *C. helgolandicus* females incubated in *P. minimum* + methanol (control condition) using REST. Data are expressed as log2 fold change.

### 4.8. Statistical Analysis

Pearson’s correlation test was performed between log2 fold change of DEGs from RNA-Seq analysis and log2 fold change of the same genes in RT-qPCR, using GraphPad Prism software v.6 (GraphPad Software Inc., San Diego, CA, USA). The same software was also used to perform Student’s *t*-test or One-way analysis of variance (ANOVA), followed by Tukey’s multiple comparison test, to evaluate statistically significant differences in log2 fold change among 10–15–20 μM PUAs MIX treatments for each GOIs.

## Figures and Tables

**Table 1:** Summary statistics of *Calanus helgolandicus* RNA-Seq analysis.

Category	Number/Length
Reads from raw data	726,490,334
Average read length (bp)	50
Reads after trimming	605,681,621
Percentage retained	83.4%
Unigenes	30,339
Average length (bp)	1427
N_50_ (bp)	1784

**Table 2:** Annotation statistics of *Calanus helgolandicus* unigenes. Nr: non-redundant protein database; SwissProt database; GO: gene onthology; KEGG: Kyoto Encyclopedia of Genes and Genomes.

Database	Number	%
All unigenes	30,339	100
Nr	21,337	70.3
Swissprot	19,386	63.9
GO	18,167	59.9
KEGG	6361	21.0

**Table 3:** Genes of interest selected from DE unigenes in *Calanus helgolandicus* females fed *Skeletonema marinoi* comparedt to females fed *Prorocentrum minimum*. Gene name, primer F and R sequences, amplicon size (As) in base pairs (bp) and amplification efficiency (E) are shown.

Name		Primer Sequence 5′–3′	As (bp)	E (%)
10-Formyltetrahydrofolate dehydrogenase	10-FTHFDH	CTTGCCAGGAACAGGAAGAG	170	111
		AGATCAGCGGAGACTTTCCA		
Betaine homocysteine s-methyltransferase 1	BHMT1	TCGTGCTGGAGCTGATATTG	143	102
		GGGTGTGATATGCCAGAGGT		
Methylenetetrahydrofolate reductase	MTHFR	TATCCACCAGGCAACACAGA	172	102
		GGCAGGATCAGCAGAAAGTC		
Vitelline membrane outer layer protein 1	VMO1	CTGGCATGAGGAACACCTTT	148	117
		AGCAGCATCCAGGTCAGTTT		
Patched domain-containing protein 3	PTCHD3	TGGAGGAATATCGGACTTGC	148	118
		TGGTGATGTCCCAGAAGTGA		
Palmitoleoyl-protein carboxylesterase NOTUM	NOTUM	TTGTACACAGGCACCAGGAA	142	117
		CACCAATGAGCACAAATTGC		
Elongation of very long-chain fatty acids protein	ELOVL	GCCCAAGATTTATTGGTGGA	192	107
		GCTGGATAGCGTGGAAGAAA		
Prosaposin	PSAP	AGACTTGGACAATTGGCTGGT	112	101
		GCACATTGTTTCCAGGTCCTC		
Pancreatic triacylglycerol lipase	PTL	CTGGCTTGAGGCTATTCCTG	179	103
		CTGAGCCTCCACTTGGGTAG		
Facilitated trehalose transporter 1	TRET1	TTTGGCTGAAAGGATTGGTC	110	92
		ACATCATCAAGGACGGGAAC		
Prophenoloxidase activating enzyme	PPAE	ATCTGCTGCCGAGTGTAACC	127	126
		TCCCCCATTATCTGCATAGC		
Microsomal glutathione s-transferase 3	MGST3	CCAGAGAGCACACCAGAACA	156	106
		GGCTCGCCTGTGTAATATCC		
Zinc finger protein OZF	OZF	TGTTTGGCTGTGAAGTTTGC	153	101
		TCAATGTGTGGGTCTTCAGG		

**Table 4:** Genes of interest selected from de novo assembled *Calanus helgolandicus* transcriptome. Gene name, primer F and R sequences, amplicon size (As) in base pairs (bp) and amplification efficiency (E) are shown.

Name		Primer Sequence 5′–3′	As (bp)	E (%)
Thymidylate synthase	TS	CCGAATACACCAACATGCAC	184	109
		TCTGCCACGTAGAACTGCAC		
Methionine synthase	MS	GGGACCTTTGATGAGTGGAA	152	95
		ACAGTGCGGCTTGTCTTTCT		
DNA (cytosine-5)-methyltransferase 1	DNMT1	ACAACAACTGGGCTGGTCTC	188	100
		GGGTGTGCCGTAGAACTTGT		
Thiamine transporter 1	THTR1	CCCGAACCAACTGTTCAAAT	189	88
		ATGGGCTGGCTTTATCTCCT		
Dihydrofolate reductase	DHFR	GATCAAGTCTGAGCTGGCGT	154	112
		CCTGGAGAGCACGATGTTGA		
Aurora kinase B	AUR	CTCAAGGAGAGCCACCATGT	193	122
		CCTCAGGTCCACCCTTGTAA		
Homeotic protein distal-less	DLL	AGTTCCCATTCCCAGGAGGT	199	97
		GGCAGAGCTAGGTACTGGGT		
Embryonic polarity protein dorsal	DORSAL	CAGCCAGCACCCAAGAGAAT	143	104
		GCATCCTTCCTTCCCAACCA		
Heat shock cognate protein 70	HSC70	TCGGAATTGATCTTGGAACC	149	103
		TGCAGCATCTCCAACAAGTC		
Sonic hedgehog protein	HH	TCTGATCTCGGACTGGTTGA	189	116
		CTGGCAGGGTAGAGAGCAAC		
Transcriptional activator cubitus interruptus	CI	TGCACGTTTGAAGGCTGTTG	153	100
		ATTCTGGTGTTTCGCCCTGT		
Protein smoothened	SMO	AATGAGGTGGAGGAGTGTGG	184	100
		AGAAGATTGCCAGAGCAGGA		
Protein Wnt-4	WNT4	GACGCACAAGACAGACGAAA	123	107
		GCACTTGCATTCAACCTTCA		
Cellular tumor antigen p53	P53	AGACCCTTCCAACAGAGCAA	186	129
		CAAGACCCGAGACACATGAA		

## Supplementary Materials

The following are available online at https://www.mdpi.com/1660-3397/18/8/392/s1. Figure S1: Size distribution of *Calanus helgolandicus* unigenes (reference transcriptome); Figure S2: BLASTx search results for *Calanus helgolandicus* unigenes against the Nr database; Figure S3: Top-Hit Species distribution of BLASTx similarity search for *Calanus helgolandicus* de novo assembled transcriptome; Figure S4: Comparative analysis of GO classifications of *Calanus helgolandicus* annotated unigenes. Table S1: Functionally annotated DE unigenes in *Calanus helgolandicus* females; Table S2: List of Gene Ontology (GO) terms for the category ‘Biological Process’ associated to up-regulated *Calanus helgolandicus* DE unigenes; Table S3: List of Gene Ontology (GO) terms for the category ‘Biological Process’ associated to down-regulated *Calanus helgolandicus* DE unigenes.
