# Supplementary material for: De Novo Transcriptome Assembly and Gene Expression Profiling of the Copepod Calanus helgolandicus Feeding on the PUA-Producing Diatom Skeletonema marinoi
Source: Mar Drugs. 2020 Jul 27;18(8):392. doi: 10.3390/md18080392 (PMC7460014; doi:10.3390/md18080392)
Supplement: Supplementary file 1 [file marinedrugs-18-00392-s001.pdf]

Supplemental Information for:

De Novo Transcriptome Assembly and Gene Expression Profiling of the Copepod Calanus helgolandicus Feeding on the PUA-Producing Diatom Skeletonema marinoi

Sneha Asai, Remo Sanges, Chiara Lauritano, Penelope K. Lindeque, Francesco Esposito, Adrianna Ianora and Ylenia Carotenuto*
Corresponding author: ylenia.carotenuto@szn.it

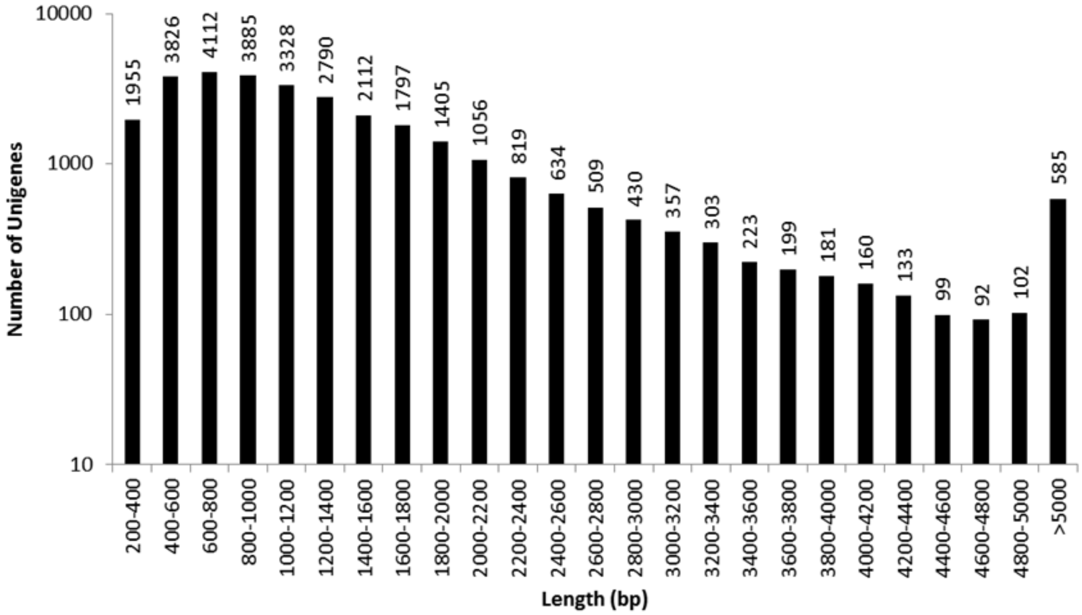

Figure S1. Size distribution of *Calanus helgolandicus* unigenes (reference transcriptome).

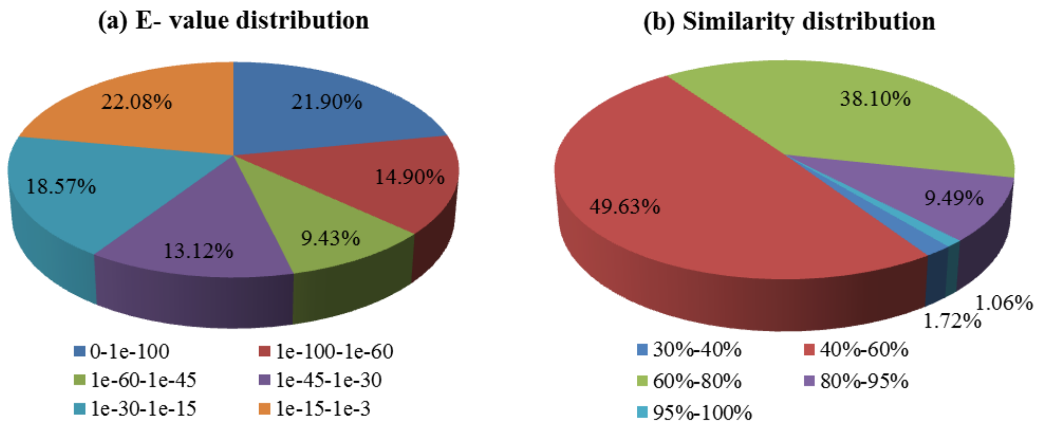

Figure S2. BLASTx search results for *Calanus helgolandicus* unigenes against the Nr database. (a) E-value distribution for each unigene with a cut-off E-value of 1E-3. (b) Similarity distribution of the top BLAST hits for each unigene.

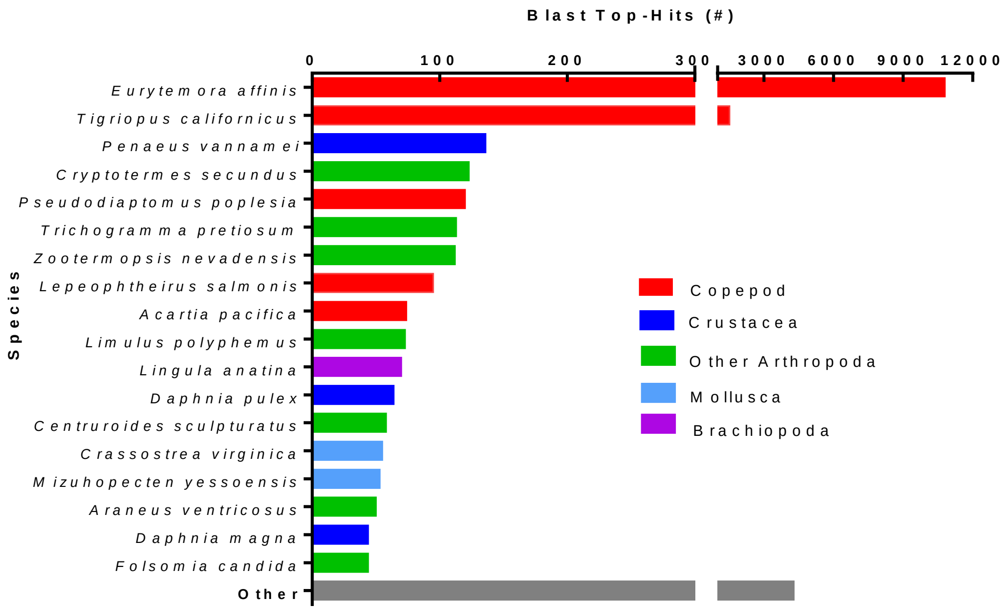

Figure S3. Top-Hit Species distribution of BLASTx similarity search for *Calanus helgolandicus de novo* assembled transcriptome. Taxonomic groups are color-coded: copepods (red), crustaceans (blue), other arthropods (green), molluscs (light blue) and brachiopods (purple).

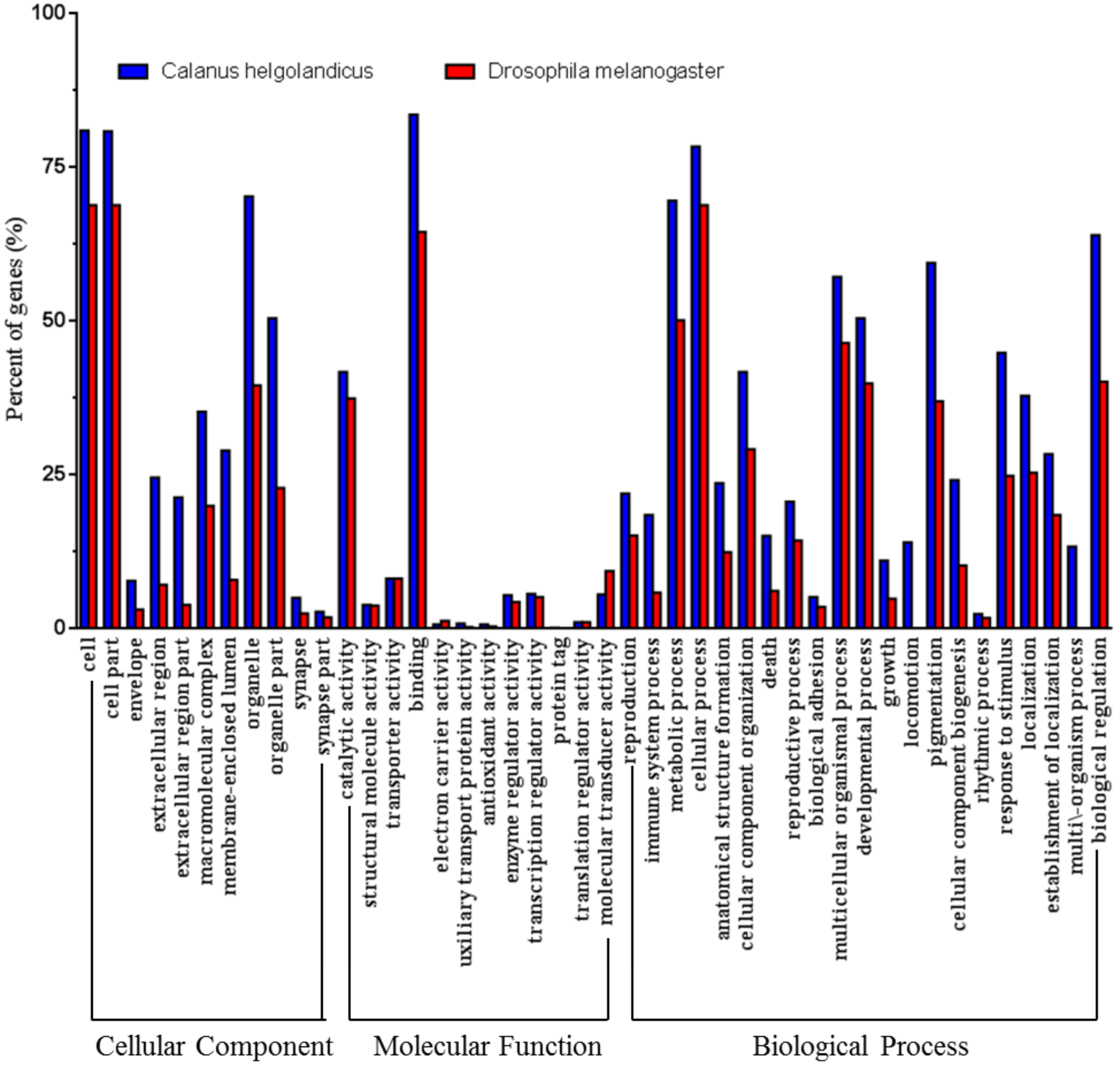

Figure S4. Comparative analysis of GO classifications of Calanus helgolandicus annotated unigenes. Percentage (left y-axis) and number (right y-axis) of unigenes assigned to top-level selected GO functional categories within the three main categories: Cellular Component, Molecular Function and Biological Process. For comparison, GO term distributions for C. helgolandicus transcriptome (red) was compared to that in Drosophila melanogaster (blue) sequenced genomes.

Table S1. Functionally annotated DE unigenes in Calanus helgolandicus females feeding Skeletonema marinoi with respect to females feeding Prorocentrum minimum. Unigene ID, protein name, length (bp), log_2 FC and FDR for up-regulated (red) and down-regulated (green) unigenes are shown.

Unigene ID	Protein name	Length (bp)	Log_2 FC	FDR
comp209668_c1_seq1	protein FAM71B-like	726	9.39	1.6E-04
comp212146_c0_seq1	zinc finger protein OZF-like	631	5.88	4.2E-02
comp216730_c0_seq1	---NA---	1554	4.99	7.1E-03
comp2182136_c0_seq1	---NA---	1548	6.47	3.5E-02
comp225760_c0_seq1	Dehydrogenase/reductase SDR family member 1	870	10.72	6.2E-03
comp225788_c0_seq1	transmembrane protease serine 9-like	1366	9.94	9.2E-05
comp226485_c0_seq2	predicted protein	1867	5.49	4.8E-10
comp236408_c0_seq1	microsomal glutathione S-transferase 3-like	542	7.47	4.8E-06
comp236721_c0_seq1	pancreatic lipase-related protein 2-like	2318	3.15	4.4E-02
comp237161_c0_seq1	ATP synthase subunit alpha, mitochondrial	452	8.88	3.1E-02
comp237332_c0_seq1	zinc finger protein OZF-like	1150	9.51	3.8E-04
comp238175_c0_seq1	E3 ubiquitin-protein ligase SINAT3	1536	2.48	3.0E-02
comp240854_c0_seq1	neuronal calcium sensor 2 isoform X1	731	1.71	1.7E-02
comp242977_c0_seq1	galactose-specific lectin nattectin-like isoform X1	602	8.24	2.4E-02
comp243955_c0_seq1	prosaposin isoform X2	410	4.16	3.9E-11
comp247017_c1_seq1	elongation of very long chain fatty acids protein AAEL008004	739	3.93	4.4E-02
comp253931_c1_seq4	heat shock 70 kDa protein cognate 4	862	1.38	3.9E-02
comp253931_c2_seq1	heat shock 70 kDa protein cognate 4	1049	1.35	3.7E-02
comp254353_c0_seq1	pancreatic lipase-related protein 2-like	2446	6.06	2.6E-03
comp255214_c2_seq1	NAD(P)-dependent alcohol dehydrogenase	1297	1.71	4.9E-04
comp255509_c0_seq1	heat shock 70 kDa protein 4 isoform X2	2880	1.82	9.4E-05
comp256916_c0_seq1	pyrimidodiazepine synthase-like	945	1.94	5.2E-03
comp257552_c1_seq1	deleted in malignant brain tumors 1 protein-like	679	1.71	3.0E-02
comp257765_c1_seq1	oncoprotein-induced transcript 3 protein-like	1038	7.19	1.6E-03
comp257885_c0_seq5	kelch-like ECH-associated protein 1	1151	1.77	2.3E-03
comp258558_c0_seq1	heat shock protein beta-1	940	1.26	4.1E-03
comp258706_c0_seq9	complement C1q tumor necrosis factor-related protein 3-like	828	1.53	2.6E-06
comp258921_c0_seq2	glycoside hydrolase family 16 protein	1045	1.05	2.7E-03
comp261379_c6_seq1	prophenoloxidase activating enzyme	1046	8.12	5.7E-03
comp261579_c1_seq3	Trypsin	751	1.11	3.5E-02

comp261981_c0_seq2	heat shock 70 kDa protein 1-like	1370	3.37	9.4E-05
comp261981_c4_seq1	heat shock protein 70	851	2.38	2.3E-03
comp262411_c0_seq2	DnaJ domain protein	442	2.96	5.3E-04
comp262411_c1_seq3	dnaJ protein homolog 1 isoform X2	400	2.90	7.7E-04
comp262678_c0_seq9	Contactin-associated protein-like 2 precursor	1536	3.86	3.9E-03
comp262718_c0_seq9	glutathione S-transferase 1-like	754	2.85	7.8E-04
comp264130_c1_seq2	Methylenetetrahydrofolate reductase	2467	1.74	1.8E-03
comp264506_c0_seq6	dnaJ protein homolog 1	614	2.45	1.4E-02
comp265753_c0_seq1	chitooligosaccharidolytic beta-N-acetylglucosaminidase	1928	1.50	6.2E-03
comp265816_c1_seq3	sequestosome-1 isoform X2	1201	3.84	1.5E-03
comp266061_c1_seq13	Contactin associated protein 1-like protein	1305	2.91	3.0E-02
comp266202_c1_seq1	carbohydrate-binding protein	391	3.80	3.7E-07
comp266202_c2_seq4	carbohydrate-binding protein	704	3.42	1.2E-09
comp266669_c2_seq7	scavenger receptor cysteine-rich type 1 protein M130-like	702	2.26	3.2E-05
comp267432_c0_seq6	heat shock 70 kDa protein 4-like	1036	3.48	6.2E-04
comp267628_c1_seq1	Niemann-Pick C1-like protein 1	3775	1.20	1.6E-02
comp267935_c4_seq1	cubilin isoform X1	2737	1.46	5.3E-03
comp269569_c0_seq1	sequestosome-1-like isoform X2	1948	1.81	1.8E-02
comp270609_c0_seq7	FAD-dependent oxidoreductase	688	1.54	4.3E-02
comp270819_c1_seq1	adhesion G-protein coupled receptor G7-like isoform X2	3214	1.29	9.0E-05
comp271888_c0_seq1	copine-8 isoform X1	2249	1.02	2.1E-02
comp272134_c0_seq1	xanthine dehydrogenase/oxidase	3740	1.41	7.3E-03
comp110100_c0_seq1	pancreatic triacylglycerol lipase-like	2325	-7.13	9.9E-04
comp120724_c0_seq1	gastrula zinc finger protein XlCGF57.1	992	-5.34	1.6E-03
comp1861472_c0_seq1	GTPase IMAP family member 4	1358	-4.71	3.2E-02
comp211093_c0_seq1	---NA---	1394	-3.27	9.9E-03
comp211525_c1_seq1	zinc finger protein 709-like	1105	-3.24	4.8E-02
comp221214_c0_seq1	---NA---	1433	-3.57	4.3E-03
comp226512_c0_seq1	ribonuclease inhibitor-like	1712	-9.68	1.7E-08
comp226882_c0_seq1	ganglioside GM2 activator-like	682	-1.65	1.3E-05
comp235997_c0_seq1	GTPase IMAP family member 7-like	1260	-6.62	4.1E-03
comp238019_c0_seq1	Chorion peroxidase	2323	-2.57	4.3E-04
comp240496_c1_seq1	---NA---	974	-3.97	2.7E-04
comp240932_c0_seq1	---NA---	1137	-1.46	2.1E-02
comp241217_c0_seq1	---NA---	764	-1.66	3.5E-02
comp241475_c0_seq1	lysosome membrane protein 2-like	1802	-3.20	6.3E-04

comp245383_c0_seq2	interferon-induced protein 44-like	2324	-10.09	2.4E-03
comp247981_c0_seq1	cathepsin L1-like	1183	-1.44	1.3E-02
comp248706_c0_seq1	kelch-like protein 17	479	-5.10	1.7E-08
comp248706_c1_seq1	hypothetical protein TCAL_06996	586	-5.10	4.1E-08
comp248933_c0_seq1	putative glucan 1,3-beta-glucosidase	1400	-1.15	2.1E-03
comp249578_c0_seq1	chymotrypsin B-like	598	-2.35	9.1E-03
comp249746_c0_seq1	AIG1 family protein	1655	-2.21	5.8E-03
comp250415_c0_seq1	probable chitinase 3 isoform X3	435	-1.27	2.0E-03
comp250520_c1_seq1	chymotrypsin-like proteinase	1083	-2.46	4.8E-10
comp250691_c0_seq1	activating transcription factor 7-interacting 1 isoform X1	1549	-2.07	3.3E-02
comp250721_c0_seq5	speckle-type POZ protein-like isoform X2	1220	-1.56	4.8E-02
comp250851_c0_seq2	facilitated trehalose transporter Tret1-2 homolog	1515	-3.13	3.0E-13
comp252102_c0_seq1	putative alpha-amylase	1561	-1.58	5.3E-08
comp252150_c0_seq1	solute carrier family 2, facilitated glucose transporter member 8-like	847	-9.43	8.6E-09
comp253678_c0_seq1	betaine--homocysteine S-methyltransferase 1-like	1532	-1.93	1.2E-06
comp253754_c3_seq19	conserved protein	1242	-3.63	9.9E-03
comp253871_c0_seq3	mucin-like peritrophin	423	-1.89	1.9E-03
comp254295_c2_seq1	dynamin-1 isoform X7	2170	-3.44	3.4E-07
comp255452_c0_seq5	nuclear transcription factor Y subunit gamma-like	925	-1.11	2.9E-02
comp255535_c0_seq1	lactonase family protein	1152	-2.30	1.4E-09
comp256514_c0_seq1	ganglioside GM2 activator-like	742	-3.11	1.7E-02
comp256660_c0_seq1	Collectin sub-family member 12	728	-1.20	1.1E-02
comp256825_c0_seq1	---NA---	438	-2.78	8.0E-04
comp256896_c0_seq1	palmitoleoyl-protein carboxylesterase NOTUM	1303	-1.62	1.0E-02
comp257329_c0_seq1	kelch-like protein diablo	1671	-1.67	1.3E-03
comp257501_c0_seq1	endonuclease/exonuclease/phosphatase family protein	1156	-2.34	5.6E-07
comp258414_c0_seq18	Glycine N-methyltransferase	1131	-1.49	2.9E-03
comp258636_c0_seq1	Endoglucanase A	3097	-1.22	1.3E-05
comp259136_c0_seq7	interferon-induced protein 44-like	2390	-1.88	3.5E-02
comp260261_c0_seq6	---NA---	766	-1.81	1.3E-03
comp260318_c0_seq2	glucoamylase precursor	1672	-1.15	2.9E-02
comp260797_c0_seq1	pancreatic triacylglycerol lipase-like	1143	-1.52	1.6E-03
comp261397_c1_seq2	endoglucanase 1	1020	-3.01	9.7E-20

comp261558_c0_seq1	betaine--homocysteine S-methyltransferase 1	1196	-1.82	3.9E-02
comp262490_c0_seq1	glycosyl hydrolase family 16	1011	-1.20	2.0E-02
comp262619_c2_seq3	facilitated trehalose transporter Tret1-like	749	-2.88	4.2E-04
comp262833_c0_seq1	endoglucanase 1	1243	-1.12	2.1E-04
comp263829_c0_seq1	glycoside hydrolase family 7 protein	1692	-1.12	2.9E-02
comp264233_c1_seq1	dimethylglycine dehydrogenase, mitochondrial	2751	-1.22	9.1E-03
comp264531_c0_seq1	nucleoside hydrolase	1685	-1.49	1.8E-02
comp264781_c1_seq1	choline monooxygenase, chloroplastic	1276	-1.57	3.7E-02
comp266018_c0_seq1	transmembrane protease serine 9-like	3164	-1.49	1.7E-02
comp266087_c0_seq4	Cationic trypsin-3	657	-2.12	3.5E-02
comp268963_c0_seq6	---NA---	1005	-1.61	2.1E-02
comp269257_c0_seq10	facilitated trehalose transporter Tret1-like isoform X1	784	-2.51	8.4E-04
comp271244_c3_seq10	cathepsin L1	1456	-1.70	4.3E-02
comp271828_c0_seq1	T9SS type A sorting domain-containing protein	1162	-4.04	1.9E-06
comp272012_c0_seq1	cytosolic 10-formyltetrahydrofolate dehydrogenase	1122	-1.36	1.2E-04
comp272012_c1_seq2	cytosolic 10-formyltetrahydrofolate dehydrogenase	1864	-1.45	3.3E-05
comp72199_c0_seq1	Plasminogen	1201	-9.06	1.9E-03
comp253223_c0_seq1	vitelline membrane outer layer protein 1 homolog	706	-4.72	4.4E-23

Table S2. List of Gene Ontology (GO) terms for the category 'Biological Process' associated to up-regulated Calanus helgolandicus DE unigenes. Level, ID, name and the number of DE unigenes for each GO term is shown.

GO level	GO ID	GO name	# DEGs
2	GO:0032502	developmental process	2
2	GO:0032501	multicellular organismal process	2
2	GO:0050789	regulation of biological process	4
2	GO:0050896	response to stimulus	5
2	GO:0065007	biological regulation	4
2	GO:0023052	signaling	2
2	GO:0048519	negative regulation of biological process	2
2	GO:0008152	metabolic process	18
2	GO:0071840	cellular component organization or biogenesis	3
2	GO:0009987	cellular process	15
2	GO:0051179	localization	2
3	GO:0009628	response to abiotic stimulus	2
3	GO:0044085	cellular component biogenesis	2
3	GO:0051234	establishment of localization	2
3	GO:0006807	nitrogen compound metabolic process	11
3	GO:0007154	cell communication	2
3	GO:0051716	cellular response to stimulus	3
3	GO:0009056	catabolic process	2
3	GO:0055114	oxidation-reduction process	5
3	GO:0048869	cellular developmental process	2
3	GO:0044237	cellular metabolic process	8
3	GO:0007165	signal transduction	2
3	GO:0009058	biosynthetic process	3
3	GO:0071704	organic substance metabolic process	15
3	GO:0050794	regulation of cellular process	4
3	GO:0006457	protein folding	5
3	GO:0019222	regulation of metabolic process	2
3	GO:0048523	negative regulation of cellular process	2
3	GO:0006950	response to stress	3
3	GO:0016043	cellular component organization	3
3	GO:0044238	primary metabolic process	12
3	GO:0048856	anatomical structure development	2
4	GO:0005975	carbohydrate metabolic process	3
4	GO:0044249	cellular biosynthetic process	3
4	GO:1901576	organic substance biosynthetic process	3

4	GO:0046483	heterocycle metabolic process	2
4	GO:1901564	organonitrogen compound metabolic process	11
4	GO:1901360	organic cyclic compound metabolic process	3
4	GO:0061077	chaperone-mediated protein folding	2
4	GO:0043170	macromolecule metabolic process	7
4	GO:0034641	cellular nitrogen compound metabolic process	4
4	GO:0006810	transport	2
4	GO:0022607	cellular component assembly	2
4	GO:0044260	cellular macromolecule metabolic process	2
4	GO:1901575	organic substance catabolic process	2
4	GO:0060255	regulation of macromolecule metabolic process	2
4	GO:0009408	response to heat	2
4	GO:0051171	regulation of nitrogen compound metabolic process	2
4	GO:0006725	cellular aromatic compound metabolic process	3
4	GO:0080090	regulation of primary metabolic process	2
4	GO:0006575	cellular modified amino acid metabolic process	3
4	GO:0044255	cellular lipid metabolic process	2
4	GO:0006629	lipid metabolic process	3
4	GO:0022411	cellular component disassembly	2
4	GO:0042026	protein refolding	2
4	GO:0019538	protein metabolic process	6
4	GO:0051186	cofactor metabolic process	3
4	GO:0031323	regulation of cellular metabolic process	2
4	GO:0006790	sulfur compound metabolic process	3
4	GO:0009266	response to temperature stimulus	2
4	GO:0006458	'de novo' protein folding	2
5	GO:0051084	'de novo' posttranslational protein folding	2
5	GO:0006518	peptide metabolic process	2
5	GO:1901565	organonitrogen compound catabolic process	2
5	GO:0009057	macromolecule catabolic process	2
5	GO:0055085	transmembrane transport	2
5	GO:0006665	sphingolipid metabolic process	2
5	GO:0006643	membrane lipid metabolic process	2
5	GO:0051085	chaperone cofactor-dependent protein refolding	2
5	GO:0043603	cellular amide metabolic process	3
5	GO:0006508	proteolysis	4
5	GO:0019438	aromatic compound biosynthetic process	2
5	GO:0006749	glutathione metabolic process	2
5	GO:1901362	organic cyclic compound biosynthetic process	2

Table S3. List of Gene Ontology (GO) terms for the category ‘Biological Process’ associated to down-regulated Calanus helgolandicus DE unigenes. Level, ID, name and the number of DE unigenes for each GO term is shown.

GO level	GO ID	GO name	# DEGs
2	GO:0008152	metabolic process	36
2	GO:0009987	cellular process	29
2	GO:0065007	biological regulation	15
2	GO:0051179	localization	5
2	GO:0050789	regulation of biological process	5
3	GO:0009058	biosynthetic process	3
3	GO:0009056	catabolic process	5
3	GO:0071704	organic substance metabolic process	3
3	GO:0044238	primary metabolic process	25
3	GO:0051234	establishment of localization	21
3	GO:0044281	small molecule metabolic process	5
3	GO:0044237	cellular metabolic process	5
3	GO:0055114	oxidation-reduction process	12
3	GO:0006807	nitrogen compound metabolic process	6
3	GO:0065008	regulation of biological quality	18
4	GO:0046483	heterocycle metabolic process	4
4	GO:0044283	small molecule biosynthetic process	3
4	GO:0006082	organic acid metabolic process	3
4	GO:1901575	organic substance catabolic process	4
4	GO:0043170	macromolecule metabolic process	3
4	GO:1901576	organic substance biosynthetic process	12
4	GO:1901135	carbohydrate derivative metabolic process	3
4	GO:0006810	transport	5
4	GO:1901360	organic cyclic compound metabolic process	5
4	GO:0019538	protein metabolic process	3
4	GO:0044248	cellular catabolic process	8
4	GO:0006520	cellular amino acid metabolic process	3
4	GO:0034641	cellular nitrogen compound metabolic process	3
4	GO:0017144	drug metabolic process	5
4	GO:0006725	cellular aromatic compound metabolic process	5
4	GO:0042133	neurotransmitter metabolic process	3
4	GO:0006790	sulfur compound metabolic process	3
4	GO:0005975	carbohydrate metabolic process	3
4	GO:0001505	regulation of neurotransmitter levels	8

4	GO:1901564	organonitrogen compound metabolic process	3
5	GO:0006544	glycine metabolic process	17
5	GO:0043603	cellular amide metabolic process	3
5	GO:1901605	alpha-amino acid metabolic process	3
5	GO:1901565	organonitrogen compound catabolic process	3
5	GO:0043436	oxoacid metabolic process	3
5	GO:0006508	proteolysis	4
5	GO:0055085	transmembrane transport	8
6	GO:0019752	carboxylic acid metabolic process	4
6	GO:0009069	serine family amino acid metabolic process	4
6	GO:0009066	aspartate family amino acid metabolic process	3
7	GO:0006563	L-serine metabolic process	3
7	GO:0006566	threonine metabolic process	3
